# Gambian cultural beliefs, attitudes and discourse on reproductive health and mortality: Implications for data collection in surveys from the interviewer’s perspective

**DOI:** 10.1371/journal.pone.0216924

**Published:** 2019-05-16

**Authors:** A. J. Rerimoi, J. Niemann, I. Lange, I. M. Timæus

**Affiliations:** 1 Department of Population Health, London School of Hygiene and Tropical Medicine, London, United Kingdom; 2 Disease Control and Elimination Theme, Medical Research Council unit The Gambia, Banjul, The Gambia; 3 Department of Public Health, Bielefeld University, Bielefeld, Germany; 4 Maternal, Adolescent, Reproductive and Child Health Centre (MARCH), London School of Hygiene and Tropical Medicine, London, United Kingdom; University of Botswana, BOTSWANA

## Abstract

**Background:**

A community’s cultural beliefs, attitudes and discourse can affect their responses in surveys. Knowledge of these cultural factors and how to comply with them or adjust for them during data collection can improve data quality.

**Objective:**

This study describes implications of features of Gambian culture related to women’s reproductive health, and mortality, when collecting data in surveys.

**Methods:**

13 in-depth interviews of female interviewers and a focus group discussion among male interviewers were conducted in two rural health and demographic surveillance systems as well as three key informant interviews in three regions in The Gambia.

**Results:**

From the fieldworker’s viewpoint, questions relating to reproduction were best asked by women as culturally pregnancies should be concealed, and menstruation is considered a sensitive topic. Gambians were reluctant to speak about decedents and the Fula did not like to be counted, potentially affecting estimation of mortality. Asking about siblings proved problematic among the Fula and Serahule communities. Proposals made to overcome these challenges were that culturally-appropriate metaphors and symbols should be used to discuss sensitive matters and to enumerating births/deaths singly instead of collecting summary totals, which had threatening connotations. This was as opposed to training interviewers to ask standardised and precise verbatim questions.

**Contribution:**

This paper presents indigenous Gambian solutions by fieldworkers to culturally sensitive topics when collecting pregnancy outcomes and mortality data in demographic and health surveys. For researchers collecting maternal mortality data, it highlights the potential shortcomings of the sibling history methodology.

## Introduction

The study of fertility and mortality is central to demography [1, 2]. Statistical data on births and deaths are always reported retrospectively, which is inherently more challenging than collecting current status data. It can lead to problems of omission and misplacement in time of events, because respondents may never have known the answer, have forgotten it or be unwilling to provide it. Certain study designs experience fewer omissions or displacement than others, for example civil registration compared to retrospective surveys, but these problems are never entirely eliminated. The nature and severity of these problems is neither the same everywhere nor entirely idiosyncratic but dependent on the context, including the cultural context.

In Africa, censuses and household surveys are the dominant vehicles for collecting demographic data, as civil registration is incomplete, particularly of deaths [3]. Data quality can be affected by almost every aspect of the research process. For example, if the information gathered relates to matters considered private and sensitive by respondents such as sexual activity, reproduction, illness and death, one could expect withholding of information [1].

What is regarded as sensitive is varied and context specific and broadly grouped into three groups of factors. First, there are questions that are considered intrusive by nature of their content, for example, about income. Second, questions where disclosure may result in harm or perceived harm to the respondent, for example about HIV status. Third, there are questions which seek to elicit socially unacceptable answers [4, 5].

In addition to the type of information being gathered, interviewers play a role in data quality and accuracy. It has been suggested that eliminating interviewers and using self-interview methods is the best way to encourage honest responses to sensitive questions[4]. Nevertheless, a large number of respondents in Africa are illiterate, and even less so computer-literate. This and attendant challenges such as power supply in remote areas leave face-to-face interviews as the most feasible mode of data collection [6–8].

Interviewer characteristics such as age, ethnicity, gender, personality, attitudes and beliefs affect survey outcomes [9–12]. Some characteristics are amenable to change through training, while others can be used to the research’s advantage, for example gender matching. Further factors, such as the language in which the interview is conducted have been studied, indicating that use of the language the respondent is most comfortable with, results in more accurate information, regardless of differences in ethnicity between the interviewer and respondent [13].

While these characteristics are not exhaustive, they give an indication of what should be taken into consideration when planning and carrying out surveys.

The cultural context in which a survey is to take place is also crucial as it may influence responses to survey questions. Understanding it provides a background against which explanations of the study outcomes can be sought [14]. Culture is a social heritage that influences people’s perceptions and responses during the process of communication. Certain cultural practices can affect data collection. For instance, post-partum confinement of new mothers and the belief that newly-born children are not yet fully human can lead to their omission by respondents, potentially resulting in underestimates of peri-or -neonatal mortality [15–18].

In The Gambia, no national surveys about pregnancy outcomes, which include stillbirths and miscarriages, and maternal mortality had been conducted since the first one in 2001 [19]. However, the country’s first demographic and health survey in 2013 utilized the sibling history to enable estimation of maternal mortality and a modified birth history to estimate stillbirth rates [20]. The reported maternal mortality rate was almost half of that estimated from the Gambian census conducted in the same year and the stillbirth rates were implausibly low [20, 21]. Neonatal mortality in the health and demographic surveillance systems (HDSS) in rural Gambia is suspected to be underestimated [22]. This paper therefore focuses on understanding cultural beliefs, discourse and practices in The Gambia that relate to pregnancy and its outcomes to identify sensitive matters for the community and to gain deeper insight into potential reasons for under-reporting in surveys. The insights are based on learning from trained and skilled interviewers, who are an excellent source of qualitative information as they are the point of contact with community members [23]. Interviewers observe the respondents’ verbal and non-verbal responses as well as their surroundings and are the direct recipients of the community’s responses as they usually hold conversations rich with information that is not recorded.

## Methods

### Study setting

The Gambia is the smallest country in continental Africa and is surrounded by Senegal except at its western border which is the Atlantic Ocean. It has a population of almost 1.9 million citizens of whom 96% are Muslim ([24]).

The Medical Research Council Unit The Gambia (MRCG) at the London School of Hygiene & Tropical Medicine runs three HDSS which cover almost 15% of The Gambia’s population. This study was carried out in Farafenni HDSS in Kerewan region on the North Bank of the river Gambia. There are three main ethnic groups–Wollof 41%, Mandinka (31%) and Fula (22%), whose primary occupation is farming [14]. As at 31^st^ December 2015, there were 55,209 individuals being followed up [25]. In this region, under-five mortality is the third highest nationally at 72/1000 livebirths, adult mortality the second lowest and fertility second highest with a total fertility rate (TFR) of 6.8 children per woman.

The Basse HDSS established in 2007 is on the south bank of the river Gambia [26]. The Serahule (39%), Fula (30%) and Mandinka (29%) are the major ethnic groups in Basse (2013 census spatial distribution). As at 31^st^ December 2015, a total of 179, 548 people were being followed up in the HDSS. Under-five mortality is 66/1000 livebirths, ranking fifth highest while the level of adult mortality is second highest nationally and has a TFR of 6.4 children per woman.

The qualitative study reported on here was embedded within two larger household surveys conducted from December 2015 to June 2016 in the two HDSS These were re-enumeration surveys of a sample of the HDSS population carried out with several objectives, one of which was to use pregnancy histories to re-estimate under-five mortality estimates including perinatal mortality. For this objective, a pregnancy history was obtained from resident women of reproductive age by female only interviewers as opposed to routine surveillance conducted by male interviewers (S1 File). Other study protocols included conditions that women were to be interviewed in private using their preferred language.

### Study participants

The first group of study participants of the qualitative study comprised 13 of the 31 female fieldworkers who conducted the pregnancy history survey (see Table 1). For the six months of the survey, the female fieldworkers were fed and accommodated in the community by the village chiefs at no cost, as is customary in The Gambia. They participated in daily activities of the community alongside the local women, including cooking, fetching water, grinding of cereals and washing, in addition to interviewing the women. The female fieldworkers each interviewed 332 women on average, although one of them interviewed 1700 women. The female fieldworkers were mostly Mandinka and Fula, which are two of three most common ethnic groups in The Gambia, the third being Wollof [27]. All fieldworkers spoke Wollof as it is the lingua franca in The Gambia.

**Table 1:** Demographic profile of the study participants.

Participants	Age	Sex	Ethnic Group	Marital Status	Education level	Fieldwork Experience
I1. interview_811_0120	30	F	Mandinka	Married	Secondary	6 months
I2. interview_811_0121	34	F	Fula	Single	Secondary	None
I3. interview_811_0122	21	F	Mandinka	Single	Secondary	None
I4. interview_811_0123	23	F	Mandinka	Single	Tertiary	None
I5. interview_811_0124	27	F	Fula	Single	Tertiary	3 years
I6. interview_811_0125 & 811_0126	23	F	Fula	Single	Tertiary	None
I7. interview_811_0127	22	F	Fula	Single	Secondary	None
I8. interview_811_0128	38	F	Mandinka	Widow	Tertiary	3 years
I9. interview_811_0130	29	F	Mandinka	Single	Tertiary	None
I10. interview_811_0131	18	F	Wollof	Single	Secondary	None
I11. interview_811_0132	23	F	Fula	Single	Secondary	None
I12. interview_811_0133	24	F	Mandinka	Married	Secondary	None
I13. interview_811_0134	26	F	Fula	Single	Tertiary	None
I14. interview_811_0140	65	M	Fula	Married	Tertiary	22 years
I15. interview_811_0141	57	M	Mandinka	Married	Tertiary	25 years
I16. interview_811_0142	48	M	Fula	Married	Secondary	17 years
FGD_I	30	M	Mandinka	Married	Secondary	5 years
FGD_II	30	M	Mandinka	Married	Secondary	4 years
FGD_III	26	M	Mandinka	Married	Tertiary	1 year
FGD_IV	29	M	Mandinka	Married	Secondary	1 year
FGD_V	33	M	Mandinka	Married	Tertiary	7 years
FGD_VI	31	M	Mandinka	Married	Secondary	3 years
FGD_VII	48	M	Mandinka	Married	Tertiary	28 years

All the female fieldworkers had completed secondary school education, and two were in university while four were enrolled in college. Most had no fieldwork experience except for three who had worked with MRC or conducted nationwide surveys before (Table 1). Although they had been born in the study areas, most of them no longer lived in the rural areas as they had left to study or work.

AJR interviewed three key informants who formed the second group of study participants, two of whom were long-serving community liaison officers and one who was a field worker having over twenty years of research experience all over The Gambia. The three key informants hail from three distinct regions in The Gambia and were selected to provide more information about cultural issues identified on analysing the in-depth interviews. The third group of study participants were six male fieldworkers and their supervisor, who live in the community and work in the HDSS, and who were responsible for follow up the residents in the HDSS both before and after the survey was completed.

### Study design

Three research methods were used: in-depth interviews of the female interviewers, key informant interviews, and a focus group discussion (FGD) with a group of male HDSS field staff. AJR developed the question guides for all the interviews and FGD after a cumulative process of information gathering during the two years she had worked in the HDSSs and lived in The Gambia. This involved observation and informal conversations about ways of communicating in the community that might affect the pregnancy history data that the female interviewers collected.

Some of the field team we interviewed had worked in both Farafenni and Basse, and thus provided different viewpoints covering the north and south bank communities. AJR did not interview the female fieldworkers as the relationship was that of employer and employees, and this power dynamic would probably have constrained their responses. The interviews of the female fieldworkers were therefore conducted by JN, who is white, and were carried out in English as all the participants were fluent. JN spent a week observing the survey procedures in the field and noted the interactions of the fieldworkers with the community members and the community’s reaction to both male and female fieldworkers. She is female and similar in age to most of the fieldworkers, which aided in building rapport.

While analysing the content of the initial interviews, AJR changed the interview guides as themes emerged that needed further investigation (S2 File). The questions focused on the experience of the female fieldworkers and their understanding of the community members’ attitude to being interviewed by a female-only team and cultural beliefs that influenced the community’s response. Recruitment was stopped after no further emerging themes could be identified by JN and AJR. AJR and JN subsequently conducted the FGD with the male HDSS field staff.

The female fieldworker interviews were conducted in Dampha Kunda and Gambisara, which are in Basse on the south bank of the river Gambia. The key informants were interviewed in Basse, Farafenni and in Fajara on the coast, where the MRCG headquarters are located. The FGD comprising the male fieldworkers and their supervisors was conducted in the Armed Forces Provisional Ruling Council (AFPRC) General Hospital in Farafenni, located on the north bank of the river Gambia.

### Sampling

Purposive selection of female interviewers was done to learn from their experience when conducting the community survey, after which the snowball technique was applied to identify key informants [28, 29]. The key informants were selected based on their knowledge of the Gambia as their main roles are to facilitate community entry for all projects by MRCG, the fact that they hailed from different regions in The Gambia, being multi-lingual, and their extensive experience doing research throughout the country. The FGD was held with all the male fieldworkers working on the Farafenni HDSS. All the in-depth and key informant interviews were digitally recorded and transcribed verbatim, using the software F4. AJR and JN made expanded field notes after the FGD and recorded their reflection on the discussion immediately after its conclusion.

### Data analysis

The analytic phase for each interview and the FGD included reading each transcript and field note, summarizing, designing tables with the contents and encoding material to emerging themes [30]. Relevant passages from the transcripts were added to the coding tables under the corresponding codes and recurring themes identified. Verification and validation of the themes through data and investigator triangulation were conducted by AJR and JN who compared their independent coding frameworks derived from responses from the different data sources and discrepancies between them were discussed and resolved[31, 32]. Both manual coding and analysis using NVIVO 10 Qualitative Data analysis software (QSR International Pty Ltd. Cardigan UK) was done.

### Ethical approval

The Gambia Government/ MRC Joint Ethics Committee and the London School of Hygiene & Tropical Medicine Ethics Committee approved the study. The authors were given the discretion when obtaining informed consent to get written or verbal consent depending on the local situation. The participants were informed of the study objectives and were reassured about anonymity and their right to withdraw at any time with no consequence. According to the participants preference and in order to obtain more frank responses, verbal consent was obtained which was audio-recorded. Permission to record the interview was also given and thus all interviews were audio-recorded. As per the AAA guidelines for informed consent, ongoing consent from the participants throughout the interviews was obtained [33]. Both AJR and JN had permits to work and conduct research in The Gambia in alignment with national regulations.

## Results

We first present Gambian cultural practices, attitudes and discourse that relate to women’s reproductive health and mortality as reported by the fieldworkers. Table 2 then summarizes the potential effects these could have on data quality and accuracy when conducting these types of surveys. It also outlines suggested solutions by the study participants to some of the challenges faced.

**Table 2:** Summary of findings, implications for surveys and proposed solutions.

Cultural practices, discourse and attitudes	How it could affect data collection in surveys	Solutions proposed
Reluctance to be counted (Fula, Serahule)	Under-enumeration and underestimation of demographic parameters	Use of inanimate objects to enumerate (for instance ask how many ‘sticks’ instead of children Enumerate singly as opposed to totals Community entry using Alkalos
Reluctance to speak of the deceased	Underestimation of mortality	
Pregnancy concealment and reluctance to speak about menstruation	Missed identification of pregnancies and their outcomes	Have women interview women directly about pregnancy and its outcomes Age matching for questions on menstruation which they termed a shared experience Use of a trusted interpreter especially as most interviewers did not speak Serahule
Stigma associated with pregnancy loss	Omission of adverse pregnancy outcomes, underestimated perinatal mortality	
Hesitance in producing legal documentation–identity and health cards (Serahule community	Poor date reporting	
Women’s position in the community -defer to heads of households for decision making -female fieldworkers preferred and accepted especially if less ‘westernized’ -women’s household responsibilities including farming and household chores	Reduced response rates -refusal by male and elderly female households to allow women in their households to participate -divorced women reluctance to reveal status Acceptance of female interviewers	In addition to individual consent from the women, permission needed to be sought from the mostly male heads of households Alkalos help in identifying households especially with extensive homonymy in The Gambia and dissolved households in the case of death or divorce Adherence to acceptable dressing norms Appropriate timing of surveys (avoid farming seasons and Ramadan) and having fieldworkers assist with chores while living in the community

### Community reluctance to be counted and to speak of the deceased

The female interviewers reported that the Fula, Wollof and Serahule exhibited a reluctance to be counted. According to one key informant (I14) of Fula origin (community liaison officer), who speaks six local languages and is familiar with the Gambian culture, this was his experience unlike with the Mandinka who did not share this reluctance to be counted.

He reported that these groups of people held on to the belief that it was not good to be counted as it had been passed down generations. He suggested that they did not know the origin of this belief but still avoided being counted even after exposure to Western education. He said:

“As far as counting is concerned, the Fula will not want to use it for humans. Instead of asking ‘how many children do you have?’ you ask, ‘how many sticks does your mother have?’ They will then respond with a number. Even animals you do not count. The history goes back even before my ancestors. They do not like to count (I14)”.

The female fieldworkers interviewed corroborated this view, saying that it was not culturally acceptable to count numbers of children because counting them would reduce their numbers, that is, increase their chances of dying (I1). It is said that, if you know how many children someone has, it enables you to curse them and cause the children’s death. A female fieldworker attributed this to the low literacy level in the study areas and thought that, over time, this concept would change as more people were educated and therefore came to understand that counting people was not directly responsible for causing their deaths (I1).

The fieldworkers encountered further resistance when asking about the respondents’ siblings, especially if the siblings were deceased, as Gambians prefer not to dwell upon those who have died (I1,I6,I7, I8). Most Fula and Serahule women interviewed refused to name their siblings. If the fieldworker probed for names, they would sometimes be told to ask their (the respondents’) parents as a way of dismissing them. In some cases, their parents were not part of the study sample, or were resident elsewhere or deceased. Often, respondents would acknowledge that they knew the number of their siblings, but some reported that they would never reveal this information, far less list their names. However, by gentle probing and by breaking down the question into two, starting by counting those who were alive first and then asking about those who had passed away, the team occasionally managed to get this information (I2).

A key informant (I15) reported that even Fula people from the neighbouring country of Guinea living in Basse have the same reticence, and that their reluctance was not related to fears about witchcraft.

### Concealment of pregnancy

Women from all ethnic groups in The Gambia, seldom talk about or discuss their pregnancies, even when they are plainly visible. A well-known proverb that justifies this was provided by a key informant as follows: “If a snake wants to grow big it has to hide.” (I14). He further explained that hiding the pregnancy was a way of protecting it from harm from others who might cause the woman to miscarry by means of the evil eye or witchcraft. For the primary survey, two questions were included to try to improve the detection of pregnancies (S1 File). The women were first asked directly by the female interviewers if they were pregnant. Regardless of the answer, they were then asked about their menstruation cycles. If a respondent reported that she had missed her period, she was further probed as to the reasons for the absence of her period while keeping in mind the sensitivity and cultural implications of revealing a pregnancy (I4). This was useful as many women readily reported missing their menstrual periods for months, but still said that they were not pregnant when asked directly. This was not always the case for older women however, as some of them felt offended at being asked about menstruation by our ‘younger’ fieldworkers.

The female fieldworkers reported that, in some instances, the woman’s husband, mother or in-laws confirmed the pregnancy, even if she would not, despite attempts at privacy by the team such as interviewing the woman under a tree out of earshot. The woman’s relatives knew that the survey was asking women about pregnancies and would tell the female interviewers that so-and-so was pregnant in spite of her reluctance to report this.

Although the women eventually responded, they found it difficult to report on dates of their last menstrual period, as asking about dates of menstruation is an unusual practice in The Gambia. The female fieldworkers said that they would sometimes encourage the respondents to speak about periods and pregnancy on the basis that these were things that all women went through (I13).

### Stigma about pregnancy losses

The pregnancy history survey (primary study) included questions on miscarriages, abortions and stillbirths (S1 File). Perhaps as a continuum of the reluctance to be counted noted previously, some women did not want to talk about their reproductive health problems and considered this information private. This was particularly so when the women were asked about pregnancy losses (I6, I7, I9, I12). The fieldworkers believed that younger women between 15 and 18 years of age found it most difficult to answer questions about pregnancy losses. For example, their standard response was that they were not married and therefore could not have had this experience (I7). This reticence is probably compounded by the fact that the community frowned upon both women having children out of wedlock and upon them having an abortion.

Although respondents were hesitant to talk about pregnancy losses, there were a few exceptions to this. Some interviewers reported that although it took a lot of time to sit and listen to the respondents, the ‘release’ reported by the respondents after talking about their pregnancy losses was worth it. This was more so as they lived in the households that sometimes belonged to the respondents (I4, I11). Unfortunately, some may have answered with the misconception that by giving out this information they would be helped to find a cure (I10). It was also difficult to capture accurate dates for stillbirths and miscarriages but not for livebirths, which were usually recorded on the child’s health card (I1, I4, I11, I13).

### Challenges with legal documentation

In the urban area in Basse, people could clearly remember the dates of demographic events, and readily produced their identification documents. In the more rural areas, gaining access to documents proved difficult.

This sentiment is echoed by (I4, I9) and (I10), who mention that refusal to produce identification documents occurred more frequently among the Serahule community, who were concerned that the interviewers would take them.

### Women’s roles and status in the community

In some villages, in addition to obtaining consent from women study participants, fieldworkers needed to first seek permission from the heads of households, who wished to determine which questions could be answered. If the household head was not around, the interviewers would have to wait or reschedule as doing otherwise meant that they could be expelled from the compound (I1). We found that most refusals were by older women or male heads of households from the Serahule community.

Also, in households where there was divorce, some of the women would not disclose that their husbands, who had been listed as the household head, had left, leading the fieldworkers to believe the women were not members of the selected households, when in fact they were (I6).

The Gambian culture dictates that hospitality offered by the community members should be accepted. The female fieldworkers were hosted at no cost for the entire six-month duration of the survey. They felt that they were well integrated and received more information because they lived in the community. They participated in daily activities including food preparation and eating from the same pot (I5). They reported that doing chores with the women reduced their workload and time pressure, which was appreciated, and they believed that this resulted in their receipt of more frank and accurate responses during the interviews. The female fieldworkers however had to adhere to dress codes that were acceptable to the communities they visited to encourage their responses. Being young, modern, educated women, sometimes they were met with disapproval when they wore ‘western’ attire such as trousers.

In The Gambia, women are required to live with their parents until they get married. For the female fieldworkers, living away from their parents was a challenge as most of them had never stayed away from their families before. Although they found this experience positive (I6, I9), they faced harsh weather conditions, and found the sleeping arrangements and food inadequate. Basse experiences the hottest temperatures in The Gambia, reaching over 40 degrees Celsius. The research team mitigated some of their discomfort by providing mattresses, insecticide-treated bed-nets, and bags of rice to supplement what the villagers provided. The hosts were also given a token of appreciation for providing accommodation.

The male fieldworkers during the FGD discussed their thoughts on the employment of female-only interviewers and what they had heard in the community about the female fieldworkers during their routine follow up visits so as to enable us to gauge the community’s attitudes to having women interviewers. According to them, female fieldworkers are the preferred choice for asking questions relating to pregnancy and menstruation as it was not culturally suitable to have men ask these questions (FGD_I-FGD_VII). They confirmed their discomfort in asking about pregnancy, so much so that they had developed ingenious ways of doing so without compromising women’s modesty. This they did, for example, by asking if the woman had a ‘yellow card’, which is the card given to women attending the antenatal clinic, thus alluding to her pregnancy without having to ask directly or make the woman respond directly. They also noted on their ledgers if the woman was visibly pregnant, thus eliminating the need to ask questions about this (usually in the late stages when it was obvious). It is important to note that male fieldworkers do not ask about menstruation during their routine HDSS rounds and they expressed great reluctance to ask a question about this if it was to be introduced, although they would do so if it were required. The male fieldworkers also felt that although the women in the community would answer questions about pregnancy when asked by men, social constraints made it difficult for them, whereas the female fieldworkers had no such constraint. Their visits to the community after the pregnancy history survey revealed that the female fieldworkers had left a good impression and the women had been happy to talk to them, stating that they were welcome to continue their surveys in the future.

### Strengths and limitations

For this study, data triangulation was applied using three approaches to qualitative data collection, comparing views from different age groups, sex and different regions in The Gambia as well as investigator triangulation. This increased our confidence in our findings, resulting in a more comprehensive viewpoint of the cultural factors affecting data collection on pregnancies, births and deaths.

Although the employment of local interviewers is often advocated, we found that having most of our interviewers from urban areas did not hamper rapport building. A limitation of this study is that community members were not directly interviewed after the female-only survey, but information was gleaned by proxy via the male interviewers who went back to talk to them. Equally, in some regards, this may be a strength of the study as the male fieldworkers are insiders in the community and more frank information might have been given to them than would have been to the researchers, if we had asked community members about their thoughts on the survey and the interviewers[34].

The female interviewers had all completed secondary education which enabled good communication between us. However, their educational level may have created a ‘space’ between them and the women in rural villages, who are unlikely to have had western education. This westernization may have affected the responses and attitudes of the community members, judging from the comments the interviewers received about their clothing. However, the fact that there were no other criticisms of, or sanctions against, the female fieldworkers from the community apart from the one occasioned by their ‘westernized’ dressing, which they subsequently corrected, suggests that it is unlikely that it resulted in significantly less information being given to them. However, inevitably, this paper represents the interviewers’ views as modulated by their own beliefs and social constructs, as well as the researchers’ interpretation based on their worldview, and may not fully depict the community’s real perspective.

## Discussion

Demographic research often studies components of culture such as behaviour, knowledge and attitudes, with the aim of making these more quantifiable in order to explain demographic phenomena such as mortality and fertility [35]. This study sought to understand how these factors influenced demographic field research in The Gambia and whether what we learned could be incorporated into data collection to enhance accuracy in view of potential underreporting of maternal, neonatal and perinatal mortality in the country [20, 21].

For research activities to be successful, Gikonyo, Bejon [36] emphasise the need to move beyond insisting on individual consent to considering the influence of the community. They note that having fieldworkers residing in the community, as well as having worked previously with the community, enhances the acceptability of a study. Similarly, it was important to have the heads of the households involved in the research process, a sentiment echoed by other researchers working in The Gambia [37]. Our study identified additional culturally sensitive methods that could improve community relations, such as having women interviewing women about pregnancy and related outcomes and menstruation, using respondents’ preferred language, employing metaphorical expressions to improve enumeration of people and vital events, and dressing appropriately. The timing of enumeration activities was key. It is important to avoid busy farming seasons and Ramadan (when people are fasting), and also to assist with chores if this was considered helpful.

In demographic and health surveys, the time needed to build rapport is lacking, making it a challenge to know whether the information that is gathered is true. Bleek [38] cites examples of glaring misinformation when the relationship between the interviewer and the interviewee is biased. There is also a need for close contact with the community to build trust, to encourage people to share confidential information willingly, rather than it being coerced from people. This is a time-consuming process, but effective in data collection for sensitive topics, and in one which could be adapted by health and demographic surveillance systems, where more time is spent by fieldworkers in the community. As this is not tenable for large-scale single-round surveys, we find that the process of data collection may be enhanced by enrolling community gatekeepers such as village chiefs and household heads to assist with entry into the community, ensuring confidentiality, use of sensitive language and adherence to dress codes. Too much emphasis cannot be placed on training of fieldworkers as well as encouraging empathy, particularly when collecting mortality data. Ultimately, the community should be involved in the whole reiterative research process, which although time and resource consuming, has been found to be most effective. However, the issue of sensitive topics, as labelled by the respondents, is more difficult to handle as they may require indirect inquiry in a circumscribed context which is often unmanageable. This then would require methodological advancement in analysis to cater for the missing information, potentially extrapolating it from closely followed populations.

The issue of concealing pregnancy for fear of harming oneself and the unborn child for example is widespread in Africa. In Mozambique and Liberia, women seek multiple protection methods to counter this ‘reproductive vulnerability’ and ensure they sail safely through the pregnancy, which is considered good fortune and thus may attract harm [39, 40]. However, the protection methods sought do not normally include western medicine as the vulnerability is considered more spiritual in nature. In the Gambian context, bad *jinne*, the evil eye and spirit creatures, which are believed to be malevolent spiritual and physical beings, are thought to target vulnerable people like pregnant women [41, 42]. Thus, a woman’s duty is to conceal the pregnancy until such a time when it is physically impossible to do so [39, 42, 43].

Pregnancy concealment has several implications for research. Conducting research that requires identification of pregnancy in the first and second trimester, for example, may go against prevailing cultural practices as it potentially exposes women to occult forces to which they feel vulnerable. On the other hand, identification of all pregnancies in a health and demographic surveillance system is essential for accurate perinatal and neonatal mortality estimates. Therefore, innovative ways need to be developed to ensure that respondents and the interviewers can provide and collect this information respectively, without compromise of their values. Thirdly, women may delay antenatal visits in a bid to protect their pregnancies, contrary to clinical recommendations that advise early visits for protection. Lastly, rapport building, and culturally appropriate measures must be applied if truer outcome measures are to be obtained.

Idioms and proverbs were used by our informants to describe the pregnant state; for example, a snake having to hide to grow big. Research in Mozambique found that pregnancy was seen as a family secret. Revealing it was compared with revealing the hiding place of the house keys, the moral of the analogy being that, if you tell people about your hiding place, you may come and find the house empty [39]. In one community in Nigeria, pregnant women were depicted as women carrying pots of water, the pot here symbolizing the womb, and water- the unborn child. To speak of a miscarriage for example, they may describe a woman slipping and losing the water with the pot intact [44]. Perhaps, the identification and application of idiomatic language when designing research instruments may be of use. Additionally, indirect methods may be employed for asking about pregnancy, such as asking a woman for her antenatal clinic card, the ‘yellow card’ as in the Gambian HDSS example.

We found that beliefs about being counted held by the Serahule, Fula and Wollof tribes, who comprise 47% of Gambia’s population, compounded by the stigma of having many deceased relatives, made data collection on mortality especially challenging [27]. Fula are also not allowed to utter certain names such as those of their spouses or their parents. Therefore, surveys that ask them to name certain members of their households go against the grain. O’Neill and colleagues noted this reticence to participate in research by the Fula who refused to have their blood taken due to a belief that their life force was getting drained [45]. We know that the Fula are numerate. Therefore, the practical implication of this reluctance to count up to the whole family’s size would be to collect a birth history which individually enumerates each child, as opposed to asking summary questions such as those on children ever born and surviving used in censuses and multiple indicator cluster surveys [46, 47].

We were met with resistance by the majority of women when soliciting them for information on pregnancy loss, due to cultural hesitance and the stigma attached to women who had experienced multiple pregnancy losses. We did find, however, that some women were comforted when given space to speak of their pregnancy losses even if they occurred in the distant past. This highlights the fact that interviews on pregnancy losses may not be unwelcome and that there may be benefits to the affected women, particularly when they interact with empathetic interviewers. In The Gambia, carrying a pregnancy to term and having a healthy child are the woman’s responsibility. Her status and security in the homestead are solidified even further if the child is a boy. On the other hand, pregnancy losses are blamed on the woman. Thus, by concealing the pregnancy and any losses, the woman protects herself from prejudice [48]. Information on this did not reach saturation, but it presents a platform for future research on the community’s, particularly women’s, wellbeing and coping mechanisms.

The combination of the community’s reluctance to be counted and pregnancy concealment carries several implications for perinatal and neonatal mortality estimation. In Senegambia, there is a confinement period before the naming ceremony (on the 7^th^ day of life) when strangers are not allowed to see new-born babies [42, 43]. These first seven days of life are critical as over 70% of neonatal deaths occur during this period in countries in which neonatal mortality exceeds 20/1000 live births, with most deaths occurring on the day of birth [49]. In Ghana, after a baby survives the first week of life, a string of white beads is given to symbolize victory through this dangerous period, and is worn for a month, after which it is changed to blue to indicate different stages of the child’s growth [50]. These practices of confinement and celebration of survival highlight a community’s awareness of the need to protect and enhance the neonate’s survival. Equally, confinement clashes with contemporary methods of protection which require that the newborn baby visits the hospital.

Currently the Islamic religious practice of almost immediate burial compounded by the community members’ reluctance to speak of the deceased could also detrimentally affect collection of neonatal and perinatal mortality data. The confinement period and lack of recognition of the neonate as an individual in this 7-day vulnerable period also serves to ‘protect’ the adults from pain in the event of death [51]. The Mandinka call newborn babies ‘angels’ who go straight to heaven when they die, and people should therefore not be sad [52].

Castle [46] describes a rich Fula family in Mali that refused to have nutritional measures taken outside their home but were amenable to be measured at home as this would avoid supernatural reprisal. Taking this into consideration to resolve the contemporary and cultural clash, postnatal home visits by health workers as recommended by WHO may remove the ‘exposure’ of the neonate to occult forces as the baby would remain confined and therefore protected [53]. These home visits may also be helpful in prevention of early neonatal deaths through early recognition of neonatal illness and referral. If death has already occurred, the visits may be useful for enumeration of these deaths.

Self-reporting is the standard form of information gathering in most surveys including the demographic health surveys[54]. Existing translation policies emphasize the need for semantic equivalence, where the sentence structure and words in the translated question are the same. However, normative equivalence, which describes the ability of the translation to address social norms is required, but often missing. Also, in many contexts, particularly in sub-Saharan Africa, not every fieldworker will speak the language of the interviewee, as was seen in our case with the Serahule community. In The Gambia, as there is no standard written format for the spoken language, use of translated verbatim questionnaires in order to improve data quality, which is often recommended, is challenging [55, 56]. In such situations, further research into what is suitable for the community and alternatives for research instrument development should be sought, keeping in mind cultural issues such as those described in this paper. Thus, ample time and resources need to be set aside for community-based research prior to the fieldwork, to properly identify these issues to avoid compromising data quality and committing injustices to the community to be studied.

Finally, to ably represent the community, the researcher is responsible for ensuring that the research is made available and used to address the struggles that the community confided in them [57, 58]. In the global health culture, the need for accurate statistics has proliferated as the numbers are used for evidence-based policy making and as a leverage for financial investment to ensure that no one is left behind [59, 60]. Statements such as ‘to count, you must be counted’ and ‘if it can’t be enumerated it won’t work’ enhance the hegemony of the quantitative. There is inherent danger in an over-emphasis on quantification, as people become neat numbers and attractive graphs that those being counted may not benefit from the counting. Thus, as we forge ahead with generating accurate statistics, the social reality of the people behind the numbers should repeatedly be brought back to the fore [59, 61–63].

## Conclusion

Throughout the demographic research process, sensitive, nuanced and responsive qualitative inquiry into the cultural context of the participants yields information on the challenges faced by researchers. This can drive adjustments to the research process based on the findings from the formative research. Although formative research was not done in this study, prior knowledge of the cultural norms concerning interactions between male and females and the likely acceptability of using female fieldworkers to ask about pregnancy and pregnancy related outcomes, and the need for community consent and community entry through the village chiefs resulted in a smoother data collection process. One valuable lesson from this study is that cultural factors influence participants’ response or non-response in surveys. Once they have been identified, such issues ought to be addressed. One example is to have women talk to women about pregnancy and menstruation. Another is to be aware that some groups are reluctant to be counted. This can be allowed for by using birth or pregnancy histories that count one by one, as opposed to summary questions on children-ever born, such as those used in censuses and multiple indicator cluster surveys. Alternatively, one can develop innovative questions that utilize idiomatic or metaphorical expressions such as ‘how many sticks’. Identifying and addressing what is considered sensitive in a community is as critical as it is context specific. This study revealed that in rural Gambia that talking about siblings was considered a sensitive topic. Sibling histories are a key method used to estimate maternal mortality, a critical health indicator, but little awareness exists among demographers that siblings can be an especially difficult group of relatives on which to collect data. Future studies should address this by conducting qualitative inquiries in other contexts into the reluctance to talk about siblings.

## Supporting information

S1 File Questionnaire–pregnancy history questionnaire. (PDF)

S2 File Guides–qualitative field research guides. (PDF)

S3 File Interviews–interview transcripts. (ZIP)
