# Supplementary material for: Gambian cultural beliefs, attitudes and discourse on reproductive health and mortality: Implications for data collection in surveys from the interviewer’s perspective
Source: PLoS One. 2019 May 16;14(5):e0216924. doi: 10.1371/journal.pone.0216924 (PMC6522014; doi:10.1371/journal.pone.0216924)
Supplement: S1 File — (PDF) [file pone.0216924.s001.pdf]

C)PREGNANCY HISTORY QUESTIONNAIRE

MRC The Gambia Unit

INTRODUCTION AND CONSENT

Hello. My name is _____________________________________. I am working with the Medical Research Council (MRC) The Gambia. We are conducting a survey of all women living in this area. The questions usually take about 20 minutes and will be mainly about your health. All of the answers you give will be confidential and will not be shared with anyone other than members of our survey team. You don't have to be in the survey, but we hope you will agree to answer the questions since your views are important. If I ask you any question you don't want to answer, just let me know and I will go on to the next question or you can stop the interview at any time.

In case you need more information about the survey, you may contact Anne Rerimoi (+2202133078).
Do you have any questions? May I begin the interview?

Signature of the Interviewer ____________________ Date ___________________

Respondent accepts ↓ Respondent refuses → END

NO.	QUESTIONS AND FILTERS	CODING	SKIP
	I would like to ask you about all the pregnancies that you have had during your life. By this I mean all the children born to you whether they were born alive or dead, whether they are still living or not, whether they live with you or somewhere else, and all the pregnancies that you have had that did not result in a live birth. I understand that it is not easy to talk about children who have died, or pregnancies that ended before full term, but it is important that you tell us about all of them, so that we can help develop programs to improve children's health.		
201	First I would like to ask about all the births you have had during your life. Have you ever given birth?	YES . . . . 1<br>NO 2	 206
202	Do you have any sons or daughters to whom you have given birth who are now living with you?	YES . . . . 1<br>NO 2	 204
203	How many sons live with you?<br><br>And how many daughters live with you?<br><br>IF NONE, RECORD '00'.	SONS AT HOME.<br><br>DAUGHTERS AT<br><br>HOME	
204	Do you have any sons or daughters to whom you have given birth who are alive but do not live with you?	YES . . . . 1<br>NO 2	 206
205	How many sons are alive but do not live with you?<br><br>And how many daughters are alive but do not live with you?<br><br>IF NONE, RECORD '00'.	SONS<br>ELSEWHERE .<br>DAUGHTERS<br>ELSEWHERE	
206	Have you ever given birth to a boy or girl who was born alive but later died?<br><br>IF NO, PROBE: Any baby who cried or showed signs of life but did not survive?	YES . . . . 1<br>NO 2	 208

PREGNANCY HISTORY QUESTIONNAIRE

207	How many boys have died?<br><br>And how many girls have died?<br><br>IF NONE, RECORD '00'.	BOYS DEAD . . . .<br><br>GIRLS DEAD	
208	Women sometimes have pregnancies that do not result in a live born child. That is, a pregnancy can end in a miscarriage, or the child can be born dead. Have you ever had a pregnancy that did not end in a live birth?	YES . . . . 1<br>NO 2	210
209	How many pregnancies have you had that did not end in a live birth?	PREGNANCY LOSSES	
210	SUM ANSWERS TO 203, 205, 207 AND 209, AND ENTER TOTAL. IF NONE, RECORD '00'.	TOTAL PREGNANCIES	
211	CHECK 210:<br><br>Just to make sure that I have this right: you have had in TOTAL _____ pregnancies during your life. Is that correct?<br><br>YES NO PROBE AND CORRECT 201-210 AS NECESSARY.		
212	CHECK 210:<br><br>NO PREGNANCY ONE OR MORE PREGNANCIES		234

PREGNANCY HISTORY QUESTIONNAIRE

212<br>Did your (last/next to last/etc) pregnancy end in a live birth, stillbirth, miscarriage, or an abortion?	213<br>Was this a single or a multipl e birth?	214<br>In what month and year (was this child born / did this pregnancy end?)	215<br>CHECK 212:<br>RECORD SAME RESPONSE	216<br>What name was given to this child? WRITE 'BABY 1' BABY 2' ,	217<br>Is (NAME) a boy or girl?	218<br>Is (NAME) still alive?	219<br>IF ALIVE:<br>How old was (NAME) on his/her last birthday?<br>**RECORD AGE IN COMPLETE [illegible]**	220<br>IF ALIVE:<br>Is (NAME) living with you?	221<br>IF ALIVE:<br>**RECORD HOUSEHOLD LINE No. OF CHILD.**	222<br>IF DIED:<br>How old was (NAME) when he/she died?<br>IF '1 YR', PROBE: How many months old was	222A<br>Were there any other pregnancies between this and the pregnancy we were just talking about?<br>IF YES, ADD IT
01<br>LIVE BIRTH .... 1<br>STILL BIRTH .... 2<br>MISCARRIAGE . 3<br>ABORTION .... 4<br>GOTO 214	SING 1<br>MULT 2	MONTH<br>YEAR	LIVE BIRTH . .... 1<br>STILL BIRTH . ... 2<br>MISCARRIAGE .. 3<br>ABORTION .... 4<br>GOTO 222A	NAME:	BOY 1<br>GIRL 2	YES ... 1<br>NO .... 2<br>222	AGE IN YEARS	YES .. 1<br>NO ... 2	HH LINE NO.:<br>GOTO 222A	DAYS ... 1<br>MONTHS 2<br>YEARS .. 3	YES .. 1<br>ADD PREGNANCY<br>NO ... 2<br>NEXT PREGNANCY
02<br>LIVE BIRTH .... 1<br>STILL BIRTH .... 2<br>MISCARRIAGE .. 3<br>ABORTION ... 4<br>GOTO 214	SING 1<br>MULT 2	MONTH<br>YEAR	LIVE BIRTH . .... 1<br>STILL BIRTH . ... 2<br>MISCARRIAGE .. 3<br>ABORTION . ... 4<br>GOTO 222A	NAME:	BOY 1<br>GIRL 2	YES .... 1<br>NO .... 2<br>222	AGE IN YEARS	YES .. 1<br>NO ... 2	HH LINE NO.:<br>GOTO 222A	DAYS ... 1<br>MONTHS 2<br>YEARS .. 3	YES .. 1<br>ADD PREGNANCY<br>NO ... 2<br>NEXT PREGNANCY

PREGNANCY HISTORY QUESTIONNAIRE

03<br>LIVE BIRTH . . . . . . 1<br>STILL BIRTH . . . . . . 2<br>MISCARRIAGE . . 3<br>ABORTION . . . . . . 4<br>GOTO 214	SING 1<br>MULT 2	MONTH<br>YEAR	LIVE BIRTH . . . . . 1<br>STILL BIRTH . . . . 2<br>MISCARRIAGE . . 3<br>ABORTION . . . . . 4<br>GOTO	NAME:	BOY 1<br>GIRL 2	YES . . . . 1<br>NO . . . . 2<br>222	AGE IN YEARS	YES . . 1<br>NO . . . 2	HH LINE NO.:<br>GOTO 222A	DAYS . . . 1<br>MONTHS 2<br>YEARS . . 3	YES . . 1<br>ADD PREGNANCY<br>NO . . . 2<br>NEXT PREGNANCY
04<br>LIVE BIRTH . . . . . . 1<br>STILL BIRTH . . . . . . 2<br>MISCARRIAGE . . 3<br>ABORTION . . . . 4<br>GOTO 214	SING 1<br>MULT 2	MONTH<br>YEAR	LIVE BIRTH . . . . . 1<br>STILL BIRTH . . . . 2<br>MISCARRIAGE . . 3<br>ABORTION . . . . 4<br>GOTO 222A	NAME:	BOY 1<br>GIRL 2	YES . . . . 1<br>NO . . . . 2<br>222	AGE IN YEARS	YES . . 1<br>NO . . . 2	HH LINE NO.:<br>GOTO 222A	DAYS . . . 1<br>MONTHS 2<br>YEARS . . 3	YES . . 1<br>ADD PREGNANCY<br>NO . . . 2<br>NEXT PREGNANCY
05<br>LIVE BIRTH . . . . . . 1<br>STILL BIRTH . . . . . . 2<br>MISCARRIAGE . . 3<br>ABORTION . . . . . . 4<br>GOTO 214	SING 1<br>MULT 2	MONTH<br>YEAR	LIVE BIRTH . . . . . 1<br>STILL BIRTH . . . . 2<br>MISCARRIAGE . . 3<br>ABORTION . . . . . 4<br>GOTO 222A	NAME:	BOY 1<br>GIRL 2	YES . . . . 1<br>NO . . . . 2<br>222	AGE IN YEARS	YES . . 1<br>NO . . . 2	HH LINE NO.:<br>GOTO 222A	DAYS . . . 1<br>MONTHS 2<br>YEARS . . 3	YES . . 1<br>ADD PREGNANCY<br>NO . . . 2<br>NEXT PREGNANCY

MRC The Gambia Unit

PREGNANCY HISTORY QUESTIONNAIRE

212	213	214	215	216	217	218	219	220	221	222	222A
Did your (last/next to last/etc) pregnancy end in a live birth, stillbirth, miscarriage, or an abortion?	Was this a single or a multipl e birth?	In what month and year (was this child born / did this pregnancy end?)	CHECK 212: RECORD SAME RESPONSE	What name was given to this child? WRITE 'BABY 1' BABY 2' ,	Is (NAME) a boy or girl?	Is (NAME) still alive?	IF ALIVE: How old was (NAME) on his/her last birthday? **RECORD AGE IN COMPLETE YEARS**	IF ALIVE: Is (NAME) living with you?	IF ALIVE: **RECORD HOUSEHOLD LINE No. OF CHILD.**	IF DIED: How old was (NAME) when he/she died? IF '1 YR', PROBE: How many months old was	Were there any other pregnancies between this and the pregnancy we were just talking about? IF YES, ADD IT
06<br>LIVE BIRTH . . . . . . 1<br>STILL BIRTH . . . . . . 2<br>MISCARRIAGE . . 3<br>ABORTION . . . . 4<br>GOTO 214	SING 1<br>MULT 2	MONTH<br>YEAR	LIVE BIRTH . . . . . 1<br>STILL BIRTH . . . . 2<br>MISCARRIAGE . . 3<br>ABORTION . . . . . 4<br>GOTO 222A	NAME:	BOY 1<br>GIRL 2	YES . . . 1<br>NO . . . . 2<br>222	AGE IN YEARS	YES . . 1<br>NO . . . 2	HH LINE NO.:<br>GOTO 222A	DAYS . . . 1<br>MONTHS 2<br>YEARS . . 3	YES . . 1<br>ADD PREGNANCY<br>NO . . . 2<br>NEXT PREGNANCY
07<br>LIVE BIRTH . . . . . . 1<br>STILL BIRTH . . . . . . 2<br>MISCARRIAGE . . 3<br>ABORTION . . . . 4<br>GOTO 214	SING 1<br>MULT 2	MONTH<br>YEAR	LIVE BIRTH . . . . . 1<br>STILL BIRTH . . . . 2<br>MISCARRIAGE . . 3<br>ABORTION . . . . 4<br>GOTO 222A	NAME:	BOY 1<br>GIRL 2	YES . . . . 1<br>NO . . . . 2<br>222	AGE IN YEARS	YES . . 1<br>NO . . . 2	HH LINE NO.:<br>GOTO 222A	DAYS . . . 1<br>MONTHS 2<br>YEARS . . 3	YES . . 1<br>ADD PREGNANCY<br>NO . . . 2<br>NEXT PREGNANCY

MRC The Gambia Unit

PREGNANCY HISTORY QUESTIONNAIRE

08<br>LIVE BIRTH ...... 1<br>STILL BIRTH ...... 2<br>MISCARRIAGE .. 3<br>ABORTION ...... 4<br>GOTO 214	SING 1<br>MULT 2	MONTH<br>YEAR	LIVE BIRTH . ....1<br>STILL BIRTH . ... 2<br>MISCARRIAGE ..3<br>ABORTION . ....4<br>GOTO	NAME:	BOY 1<br>GIRL 2	YES .... 1<br>NO .... 2<br>222	AGE IN YEARS	YES .. 1<br>NO ... 2	HH LINE NO.:<br>GOTO 222A	DAYS ... 1<br>MONTHS 2<br>YEARS .. 3	YES .. 1<br>ADD PREGNANCY<br>NO ... 2<br>NEXT PREGNANCY
09<br>LIVE BIRTH ...... 1<br>STILL BIRTH ...... 2<br>MISCARRIAGE .. 3<br>ABORTION .... 4<br>GOTO 214	SING 1<br>MULT 2	MONTH<br>YEAR	LIVE BIRTH . ....1<br>STILL BIRTH . ... 2<br>MISCARRIAGE ..3<br>ABORTION . ... 4<br>GOTO 222A	NAME:	BOY 1<br>GIRL 2	YES .... 1<br>NO .... 2<br>222	AGE IN YEARS	YES .. 1<br>NO ... 2	HH LINE NO.:<br>GOTO 222A	DAYS ... 1<br>MONTHS 2<br>YEARS .. 3	YES .. 1<br>ADD PREGNANCY<br>NO ... 2<br>NEXT PREGNANCY
10<br>LIVE BIRTH ...... 1<br>STILL BIRTH ...... 2<br>MISCARRIAGE .. 3<br>ABORTION ...... 4<br>GOTO 214	SING 1<br>MULT 2	MONTH<br>YEAR	LIVE BIRTH . ....1<br>STILL BIRTH . ... 2<br>MISCARRIAGE ..3<br>ABORTION . ....4<br>GOTO 222A	NAME:	BOY 1<br>GIRL 2	YES .... 1<br>NO .... 2<br>222	AGE IN YEARS	YES .. 1<br>NO ... 2	HH LINE NO.:<br>GOTO 222A	DAYS ... 1<br>MONTHS 2<br>YEARS .. 3	YES .. 1<br>ADD PREGNANCY<br>NO ... 2<br>NEXT PREGNANCY

PREGNANCY HISTORY QUESTIONNAIRE

MRC The Gambia Unit

NO.	QUESTIONS AND FILTERS	CODING CATEGORIES	
223	When did your last menstrual period start?<br><br>(DATE, IF GIVEN)	DAYS AGO .... 1<br>WEEKS AGO .... 2<br>MONTHS AGO .... 3<br>YEARS AGO .... 4<br>IN MENOPAUSE/<br>HAS HAD HYSTERECTOMY ... 994<br>BEFORE LAST BIRTH .... 995<br>NEVER MENSTRUATED 996	
224	Are you pregnant now?	YES .... 1<br>NO .... 2<br>UNSURE 8	
225	How many months pregnant are you?<br><br>RECORD NUMBER OF COMPLETED MONTHS.	MONTHS	
