# Supplementary material for: Gambian cultural beliefs, attitudes and discourse on reproductive health and mortality: Implications for data collection in surveys from the interviewer’s perspective
Source: PLoS One. 2019 May 16;14(5):e0216924. doi: 10.1371/journal.pone.0216924 (PMC6522014; doi:10.1371/journal.pone.0216924)
Supplement: S2 File — (PDF) [file pone.0216924.s002.pdf]

D) QUALITATIVE RESEARCH FIELD GUIDES

v1.3 FIELDWORKER INDEPTH INTERVIEW GUIDE
MRC UNIT, THE GAMBIA
MARCH 2016

Socio-demographic information
1). Where are you from in The Gambia?
P1: Where do you live right now?
P2: How long have you been there? Migrated?
P3: Do you originate from this village?
Relationship with community members
2). How would you describe your relationship with the other members of the community?
P1: Is it a good relationship?
P2: Is it a bad relationship?
3). How did the community react on your new responsibility?
P1: What is your impression?
P2: Did your being female have any influence on the responses from the community? How?
Are there certain people who find it more difficult (men, young ladies, very old ladies, people from a certain ethnic group)? Why do they find it difficult?
P3: Do you feel it is difficult for some women to tell you about their health information? If yes:
Why do you think it is difficult for them?
Are there certain people who find it more difficult (young ladies, very old ladies, people from a certain ethnic group)? Why do they find it difficult?
Fieldwork experience
4). Please tell me about your experiences during this fieldwork
P1: What do you think went well?
P2: What were the challenges?
P3: Did you have any positive experiences?
P4: Did you have any negative experiences?
P5: Do you have a suggestion about how this could be solved?

Socio-demographic characteristics
Which ethnic group do you belong to?
How old are you?
What is your marital status?

V1.3 FIELDWORKER FOCUS GROUP DISCUSSION GUIDE

MRC UNIT, THE GAMBIA
MARCH 2016

Socio-demographic information

1). Where are you from in The Gambia?
P1: Where do you live right now?
P2: How long have you been there? Migrated?
P3: Do you originate from this village?

Relationship with community members

2). How would you describe the female team's relationship with the other members of the community?
3). How did the community react to them?
P1: What is your impression?
P2: Do you think their being female had any influence on the responses from the community? How?

Are there certain people who find it more difficult (men, young ladies, very old ladies, people from a certain ethnic group)? Why do they find it difficult?

P3: Do you feel it is difficult for some women to tell you about their health information? If yes:

Why do you think it is difficult for them?

Are there certain people who find it more difficult (young ladies, very old ladies, people from a certain ethnic group)? Why do they find it difficult?

Fieldwork experience

4). Have you conducted any interviews in the community after the female only team left?
P1: Can you describe your different experiences between the interviews before and after?
What was an especially good/bad interview? And where was the difference?

Socio-demographic characteristics

Which ethnic group do you belong to?
How old are you?
What is your marital status?
