# Supplementary material for: Gambian cultural beliefs, attitudes and discourse on reproductive health and mortality: Implications for data collection in surveys from the interviewer’s perspective
Source: PLoS One. 2019 May 16;14(5):e0216924. doi: 10.1371/journal.pone.0216924 (PMC6522014; doi:10.1371/journal.pone.0216924)
Supplement: S3 File — (ZIP) [file pone.0216924.s003.zip › S3_interviews/interview_811_0120.pdf]

Interview One

Setting: Gambakunda, in a courtyard in front of a house of a respondent

Date: 16.03.2016

Time: 13:58

Total interview time: 08:32

I: Now I will come to some questions about your relationship with community members. How would you describe your relationship with the other members of the community? #00:00:59-1#

P: The relationship is is fine anyway because we are trying to speak the languages but ahm anyway is fine yeah because the language barrier somehow but we try to manage it #00:01:17-9#

I: So it is a //good relationship? Yeah// #00:01:22-3#

P: //It is a good relationship yes// #00:01:23-4#

I: How did the community react on your new responsibility? #00:01:29-6#

P: The community we visit or? #00:01:32-8#

I: Yeah #00:01:33-0#

P: Yes some villages is difficult because when we come you have to wait for the household head before you can conduct anything they and there are certain questions some male households do not allow us to ask. Really if we start before the sitting (inc.) from the they will come they will tell you: "go out". When we have this problem we have to leave the compound. We do encounter but. #00:02:07-8#

I: Did you your being a female had any influence on the responses from the community? #00:02:17-0#

P: No #00:02:18-6#

I: No #00:02:19-3#

P: No #00:02:19-6#

I: Okay, do you fell it is difficult for some women to tell you about their health information? #00:02:26-2#

P: Yes the older women or people that are older that was like modest age and is (inc.) some somehow sometimes they not go but we do try to get something from them. #00:02:41-9#

Commented [J1]: Good Relationship to community

Commented [J2]: Language Barrier

Commented [J3]: Language Barrier

Commented [J4]: Good Relationship to Community

Commented [J5]: Problems before Interview

Commented [J6]: Problems get Information

Commented [J7]: Influence being female

Commented [J8]: Difficulites Health Information

I: So now we will get to your field experiences #00:02:46-2#

P: Yeah #00:02:46-7#

I: Please tell me about your experiences during the fieldwork #00:02:51-3#

P: Yeah you meet with different people especially the 21, we have we have been friends now and the community we visit like this lady we the/ (inc.) we come here she gives us food free we do have some very nice people in the community but is different somewhere people are not the same. We find nice people to placed and on the other on the other hand you have a different person ((laughing)). But is nice working with the community (inc. (Yes they are really nice.)) #00:03:29-2#

I: What do you think went well? #00:03:36-2#

P: Yeah? #00:03:37-7#

I: Generally what do you think went well? #00:03:41-0#

P: Generally (.) is a challenge. And I have met with different people anyway. I think I have better experience now being field/ female fieldworker. Yeah. #00:03:56-5#

I: Did you have any positive experiences? #00:04:01-5#

P: Yeah like to approach people when you are good the community how to approach them how to comport yourself I know (inc.). You know you just have to go the right question you just have to cool yourself down (inc. (first question handle)). Yeah. #00:04:22-1#

I: Did you have any negative experiences? #00:04:25-6#

P: Yeah. (Laugh) I do, yes when you are out they tell you also that (inc.) and they come like the distance from Basse you go in a car is difficult. Spend the night on the floor, go and and (inc. (find)) a room. Yeah. I really have a problem at my chests, side pain. The first 18 days that we were in. Yeah. That has been difficult. #00:05:01-3#

I: Do you have any suggestion how about how this could be solved? #00:05:08-2#

P: Yes the suggestion I think is they should find a good accommodation for us. For us to delivered while there, because it is not easy going to an accommodation that day, you don't know anybody there (inc.) no fried spending the night on the floor you take care of your food your health everything. A distance far away from where you stay. They should do something. Yeah. #00:05:36-0#

I: Can you remember the first and the last interview interview that you performed? #00:05:44-3#

P: Yeah our first interview was in Basse Basse Santa su. The last one was here in

Commented [J9]: Good Relationship between Fieldworkers

Commented [J10]: Good Relationship to Community

Commented [J11]: Enjoy work

Commented [J12]: Meeting different people

Commented [J13]: Better experience in field

Commented [J14]: Approach/Encounter People

Commented [J15]: Comfort with situation

Commented [J16]: Difficulties Transport

Commented [J17]: Difficulties Sleeping and Accomondation

Commented [J18]: Health Problems

Commented [J19]: Better supply in field

Commented [J20]: Better supply in field

Gambakunda (inc.). ((laughing))#00:05:55-6#

I:((laughing)) Can you describe the difference the different experiences between the interviews? #00:06:02-8#

P: Yeah. Interviewing here the household list was too long. The once in Basse are not that much. Yeah. And the languages are different. Basse you speak Mandinka and Fula but here majority speak Serahule. #00:06:17-0#

Commented [J21]: First interview

Commented [J22]: Last interview

Commented [J23]: Last interview

I: Okay #00:06:18-4#

P: Yeah #00:06:18-6#

I: What was an especially good and bad interview? And where was the difference? What make like the difference between them? #00:06:30-4#

P: (...) To get the papers like the ID-Card, the clinic cards is difficult around here. But in Basse areas varies. Yeah. Somebody will tell you date of birth, the educational level. But here you have to wait or you have to go and come back to get the documents from them. Is really difficult in this area. #00:06:57-5#

Commented [J24]: Bad interview

Commented [J25]: Bad Interview

I: What where the questions you found most difficult to ask? #00:07:04-3#

P: Yeah ((laughing)). Menstrual Cycle and women's capture name and the household lists. The age of the (inc. , unclearly spoken) they are very old but they not allow you to know their exact date of birth. Yeah. And the women's capture name is hard asking them. How many births do your mother have? They say "no good to my mother. Ask my mother these questions" ((laughing)). Those are basically the difficult questions. #00:07:38-5#

Commented [J26]: Most difficult question

Commented [J27]: Most difficult question

Commented [J28]: Most difficult question

I: What questions do you feel the respondents found hard to answer? #00:07:44-9#

P: Yeah. They will doubt how many how many do your mother have, just siblings. They will doubt it. Most villages they will say: "I do not know. Even if I know I will not tell you!" Yeah. #00:07:59-3#

Commented [J29]: Most difficult to answer

I: Okay, we are nearly at the end. So I have some/ At the end some socio-demographic characteristics from you. So you only answer them if you want. #00:08:11-4#

P: Yeah #00:08:11-9#
