# Supplementary material for: Gambian cultural beliefs, attitudes and discourse on reproductive health and mortality: Implications for data collection in surveys from the interviewer’s perspective
Source: PLoS One. 2019 May 16;14(5):e0216924. doi: 10.1371/journal.pone.0216924 (PMC6522014; doi:10.1371/journal.pone.0216924)
Supplement: S3 File — (ZIP) [file pone.0216924.s003.zip › S3_interviews/interview_811_0121.pdf]

Interview Two

Setting: Gambakunda, in a courtyard in front of a house of a respondent
Date: 16.03.2016
Time: 14:11
Total interview time: #00:15:53-7#

I: Ahm so now I will ask you some questions about your relationship with the community //members// #00:00:44-8#

P: //Okay// Okay no problem #00:00:46-1#

I: Ahm how would you describe your relationship with the other community members? #00:00:51-0#

P: Ah ah (...) What do you mean? The community in the community where I am staying? #00:00:57-7#

I: No, when you come to the villages. #00:00:59-4#

P: Ah okay. I just ah describe myself just like they themselves, because if you go to a certain community the the way you meet their moving just the way you also used for them. YES. You also meet them their their (inc. , unclearly spoken (least time)) the way you meet them with their (inc. , unclearly spoken (least time)) if you want to go with them you have to follow them. Yes. You go with them, bit by bit until you understand it all. Yes. #00:01:29-5#

I: Ahm It/ Is it a good relationship? #00:01:32-4#

P: YES! They are good. #00:01:36-8#

I: Generally it is a good relationship //you would say//? #00:01:37-3#

P: //Yeah// #00:01:36-6#

I: Okay. How did the community react on your new responsibility? #00:01:43-6#

P: (...) Pardon? #00:01:46-2#

I: Ahm the responsibility that you have with the ah survey? #00:01:50-8#

P: Yeah #00:01:51-1#

I: How did the community react to this? #00:01:54-0#

P: Yeah. You know some of them they are (inc. , unclearly spoken). They are not that much they don't go they don't went go to school, they are not educated. Before they understand you, you have to/ its a little bit difficulties because some of them feel you bother them, you explain yourself to them. It's at the beginning they don't want to understand. You have to encourage them, you have to encourage them until they (inc.) they/ you/ they'll und/ (inc.) they'll understand it yes. Because sometimes they don't want to cope with people @(.)@

yes. When we ask them certain questions they don't want to understand those questions. But you have to/ people like me I just take it easy easy with them until they understand. Yes. #00:02:41-8#

I: Ahm. What is your impression? #00:02:47-3#

P: With the with the with the community? YES! They are nice, they are nice they are so nice. Yes. #00:02:54-5#

I: Ahm. Did your being a female had any influences on the responses from the community? #00:03:03-0#

P: Yeah. You know some of them the used the used to/ some of them, when you asked them their menstural cycle it is very difficult. And their siblings. That one is very difficult. They cannot tell you the number of children their mothers have or their menstrual (...) cycle. Is always difficult. When we arrive at that point you will ask them (inc. , unclearly spoken). When you reach at that point they say: "no". They are not will to answer that questions. Yes yes those questions, they are not willing to answer them. #00:03:37-8#

I: Do you feel it is difficult to some women to tell you about their health information? #00:03:44-1#

P: Ah the the the the the the the the community? #00:03:47-4#

I: Yeah. The fe/ ahm the women you have been interviewing? #00:03:51-2#

P: Yes Yes. #00:03:51-7#

I: Was it difficult for them to answer some health health information questions that you have asked in the interview? #00:03:58-1#

P: YES! Especially what I am telling you, especially the menstrual cycle. Very (inc.) there. And the siblings. Is always difficult //mhm// for them to answer these questions. And the number of the miscarriage, yes. The miscarriage and the number of children tho the women they themselves have. Because you know it is a taboo. Some of them they think that as a taboo you can't the number of children they have. Yes @(.)@. #00:04:29-0#

I: Why do you think it is difficult for them? #00:04:34-3#

P: Ah it is because, it is because they/ they are they are at to my suggestion. Because they are they are not educated, but (inc. (extend)) that's why. They don't have the understanding. Yes they think ah in by counting their number children, that will make a reduce. Yes, that's their concept. That's why. But as time goes on they UNDERSTAND. We have some of them now they are UNDERSTANDING. Because if you explain it to them, some of them they have understand. Yes @(.)@. #00:05:10-0#

I: Okay now we will continue with your general work experiences, ah field experiences that you have. Ahm. Please tell me about your experiences during the fieldwork. #00:05:21-0#

P: Ah, yes. I experienced (.) many things. I experienced different people, community and the language. Because, you know this people they cannot speak, äh the language they speak/ They are Sarahules. But some of them, I thank god, because I can speak ah I can speak sur Sarahule, I can speak Mandinka, I can speak Fula, I can speak Wolof. So some of them/ it for example if you contact this ah comu/ compound here they are Sarahules. But some of them can speak Mandinka and me I have the opportunity that I can speak Mandinka. Yes. (...) I have that opportunity. #00:06:01-2#

I: Do you want do a add anything? Do you want to add something (.) to this question? #00:06:06-1#

P: Yes ah (inc.) our ah stay. Because when we stay, there was a time we are stay in Bararay for three weeks. It was really difficult for our for us. Because there our mattress and our feeding it was a problem. This a (inc.) from that place because were we slept that mattress was not that it is only few inches. Yeah, that was my problem. Apart that and the feeding. Yes #00:06:40-4#

I: So what //@(.)@// (kids screaming in the background) #00:06:44-0#

P: //@(.)@// #00:06:44-8#

I: What do you think went well? #00:06:46-9#

P: Pardon? #00:06:46-8#

I: Ah what do you think went well? #00:06:50-2#

P: Ah ah, during during our stay? YES #00:06:53-6#

I: General #00:06:54-3#

P: Generally? YES there are things that are going because,w e are coping with the community. (inc.) then those fieldworkers are helping us when we are come to the village. We we we when we talk to them some of them they understand. Yes, we are going through little bit small small, yes. Because do you know you cannot force them you cannot force them. You just have to make them you understand, it is order you get what you want from them. Yes you have to encourage so that you you can get what you want from them. You cannot (inc.) and you cannot fo-force them. Yes you have to take things easy easy, so that you get what you want from them, yes. Because we we are the one we are the one we we are the one who leaves something from them. So you have to take things easy easy and we explain to them so that they can have understanding. Yes, so that at the end of the day you can get what you want @(.)@. #00:07:58-0#

I: @(.)@ What were the challenges? #00:08:01-4#

P: Haaa. In the field? Yes. Ah the challenges. (.) The language the language äh the language barriers. Yes. And this the the the the the the the siblings thats one is our problem. The siblings is our problem. This people they are not ready. Yes, they are not ready to answer

those questions. #00:08:33-8#

I: (...) Did you have any positive experiences? #00:08:37-2#

P: YES, yes I have some, because as a fieldworker, I have not I have never been in the field. But now I have the EXPERIENCE. Yes, even ah for example when I when now when I am posted or when I have the opportunity opportunity to get with you I have already I already have the experience. So I know now how to work with the communities yes. Because I know the type of people, I have met with all type of people. Now I I can I can see that I know the community, and I can go with/ I can move. When I am posted in any community, I can go with them. Yes @(.)@ #00:09:20-0#

I: @(.)@ Did you have any negative experiences? #00:09:22-9#

P: Negative experience @(.)@ Yes yes. Ah that’s the only the time when we were aah (...) posted in when we are camping in Bararay. Yes. There was water problem, there is no electricity, no water and the the the our mattresses ah yeah with that and our feeding. Yes, that was a problem, but apart from that no. I thank god I have positive I have negative, but I have positive too. Yeah. #00:09:54-6#

I: Ah do you have a su-suggestion how the negative problems could be solved? #00:10:00-1#

P: Yes yes, we are we are (inc.) they can help us yes. Our negative ah problem I just begging them their (inc.) if they can help us, with the if they can give us a he-helping hand, when we are in ah the place. Yes. Our feeding the the aaaaaaa accommodation, our mattresses, yes. #00:10:31-1#

I: Can you remember the first and the last //@(.)@// interview you performed? #00:10:38-6#

P: //@(.)@// The first yes. The first interview ah was a a a women (inc.) until I reach at the menstrual cycle she said "no, she will not answer those question". Yes, she said. And I asked her about siblings, she let me not ask her about those things. She had told me what she can told me. That one is (inc., unclearly spoken and kids loud in the background) but I have to beg her, have to comfort her, after yes I after I thank god I get what I want from her, after comforting and begging her, at the end I get what I want from her. But at at the first time, she said "no" @(.)@. They no don’t ask those questions. And I have to explain things to her, things thi/ after she understands, she understood yeah okay. #00:11:38-4#

I: And the last interview? #00:11:43-1#

P: The last interview #00:11:44-3#

I: That you had? #00:11:45-5#

P: @(.)@ Today or? #00:11:48-3#

I: @(.)@ yeah #00:11:49-0#

P: Ah the last YES. That one was not, there was no problem yes. I interview the the the women. After the end there was no problem, yes. #00:12:01-8#

I: Ah what was an especially good and a especially bad interview? And where was the difference? #00:12:10-2#

P: The difference #00:12:11-4#

I: Yeah #00:12:12-1#

P: The difference was ah the the the the the the the the the good the good interview was, I interviewed her about all her children, the miscarriage, the stillbirths, she has given me all those things. PERFECT. Yes, but at the end, when I arrive at the siblings, she says she don't know she don't know, the number of children the mother has. Yes. After she said, because some of them has passed away, she don't know those people. Now I have to ask her "Now you don't know those who passed away, but maybe you can hear you have heard about them, even you don't know them, you can heard about them. And those who are alive." I asked her "How many of them are a-alive?" She told me and after I asked her "How many how many of them has passed away, she told me those one and that was the time, when I get what I want from her. Yes @(.)@. #00:13:16-5#

I: Ahm, what were the questions you found most most difficult to ask? #00:13:22-7#

P: Ah from the that's menstrual cycle and the siblings yes, those are the only difficult questions. Yes. And the miscarriage, the stillbirth and the miscarriage. Some of them even though they have, when we ask them they will say "no". Maybe until when the husband came, some of them their husband used to some of them, when we asked them some of them, they used to forget. Yes. Some of them/ you know they they would not know it , they they used to forget so I but when asked them, you asked keep on asking them asking them, after the used they they they they remember. Some of them they used to remember. Yes, they say "Ah yes I had a miscarriage. You tell this this time of the year." Yes. #00:14:12-0#

I: What questions do you feel the respondents, so the people you interviewed/ #00:14:18-6#

P: Yeah #00:14:18-7#

I: ah found hard to answer? #00:14:22-2#

P: That is the mh ah mhm the the the the the miscarriage, yes, the miscarriage and the menstrual cycle @(.)@ those one. Those one @(.)@ are very difficult for them to answer those questions #00:14:35-9#

I: //So// #00:14:37-0#

P: //But// still we try still we try our best. yes yes #00:14:41-2#
