# Supplementary material for: Gambian cultural beliefs, attitudes and discourse on reproductive health and mortality: Implications for data collection in surveys from the interviewer’s perspective
Source: PLoS One. 2019 May 16;14(5):e0216924. doi: 10.1371/journal.pone.0216924 (PMC6522014; doi:10.1371/journal.pone.0216924)
Supplement: S3 File — (ZIP) [file pone.0216924.s003.zip › S3_interviews/interview_811_0122.pdf]

Interview Three

Setting: Gambakunda, in a courtyard in front of a house of a respondent
Date: 16.03.2016
Time: 14:28
Total interview time: #00:07:05-7#

I: Okay (.) ahm (.). So, now I will come to the relationship with the community members you have been working with. #00:00:34-5#

P: mhm #00:00:34-8#

I: Ahm how would you describe your relationship with the other community members? #00:00:40-8#

P: Other community members? #00:00:43-7#

I: The community members, your relationship. #00:00:45-7#

P: Ah those we are working with? #00:00:48-5#

I: Yeah #00:00:48-8#

P: It's so they they are friendly to us. Yeah #00:00:56-9#

I: So it is a good relationship, //generally//? #00:00:59-8#

P: //Yeah// yeah #00:01:00-1#

I: Ahm how did you/ how did the community react on your new responsibility? #00:01:07-7#

P: Mhm? #00:01:08-0#

I: So, you got/ ahm how did the community react to to your responsibility trough this survey you have been doing with the interviews. How did the community re-react to this? #00:01:20-8#

P: Yeah, sometimes it is just difficult. Ahm when we went to some places, villages when we went there, we will ask them for compounds, sometimes they will not come out, sometimes some will come, but some will not come out to interviewed, to be interviewed. (inc.) #00:01:38-7#

I: So, what is you general impression? #00:01:42-1#

P: Mhm? #00:01:42-5#

I: What is your general impression of the fieldwork? #00:01:46-0#

P: Fieldwork is hard (...) is very hard @(.)@. #00:01:51-2#

I: Did your being female had any influence on the responses from the community? #00:01:59-8#

P: What? #00:02:00-5#

I: Ahm, did your being female had any influence on the community? So #00:02:07-4#

P: No #00:02:10-9#

I: No, not that you noticed? Ahm, do you feel, that it it is difficult for some women to tell you about their health information? #00:02:18-7#

P: Yeah, is difficult for them some. Yeah #00:02:21-8#

I: Ah why do you think it is difficult for them? #00:02:26-4#

P: I think they feel (inc., speaking to quite and kids screaming in the background) yeah. #00:02:32-0#

I: A-Are there certain people who find it more difficult than others? Like old women, young women? #00:02:39-0#

P: Ah what? #00:02:40-2#

I: Are there certain people who find it more difficult than others? #00:02:44-0#

P: Ah? #00:02:45-1#

I: The people you have interviewed, are dif/ were there differences, like where there maybe old older women who find it more difficult to answer health information? #00:02:56-8#

P: Ah, the elderly people. #00:02:59-1#

I: Yeah, the elderly people, did they find it more difficult //or//? #00:03:04-4#

P: //Yeah// Yeah Yeah #00:03:04-0#

I: Yeah, so now I will continue with the general field experiences that you had. @(.)@ #00:03:10-8#

P: @(.)@ mhm #00:03:10-9#

I: Please tell me generally ahm about field ahm about your experiences during the fieldwork #00:03:19-4#

P: Generally? #00:03:20-1#

I: Yeah, everthing. #00:03:22-0#

P: Everthing? @(.)@ #00:03:22-7#

I: @(.)@ That you want to tell me. #00:03:25-2#

P: Yeah, fieldwork I/ is hard, when you interview you have some problems like feeding, when we went to the villages at Bakaday. Yeah, some problems on the feeding, we spend most of the time, we spend most of our morning on feeding. Yeah #00:03:48-4#

I: Ah what do you think went well? #00:03:51-5#

P: Mhm? #00:03:52-1#

I: What went well? #00:03:54-0#

P: What? #00:03:54-6#

I: What ah were good experiences in the fieldwork? #00:03:58-8#

P: What am I going to? #00:03:59-8#

I: What were good experiences in the fieldwork, generally, good ones that you had? #00:04:06-3#

P: Go to the next question (...) go to the next question #00:04:10-6#

I: Okay #00:04:11-1#

P: I don't //understand// #00:04:11-7#

I: //Okay, yeah// Where were challenges for you? #00:04:15-4#

P: Yes @(.)@ during, yeah we were in the field one day, I got/ I fell down on the ground, I got some bruses on my //arm//. Yeah yes this time. @(.)@ #00:04:31-7#

I://mhm// ahm, did you have any positive experiences? #00:04:35-8#

P: Positive? Yeah I I am interacting with the community here. I am just allowed to be with people right here, yeah #00:04:47-1#

I: Ah did you have any negative experiences? #00:04:49-9#

P: Negative? No. From People? #00:04:53-5#

I: Yeah, generally #00:04:54-8#

P: Generally? (inc, speaking to quit and to much noise in the background) #00:04:58-3#

I: Ah, do you have a su-suggestion, an idea, how the difficulties could be solved? #00:05:08-7#

P: Even, if they can help us with food, when we are in the village, yeah that will be better. #00:05:16-0#

I: Okay (...), ah can you remember the first interview and the last interview that you performed? #00:05:23-5#

P: Can I remember the first one? @(.)@ The first/ yeah yeah, yes I can remember. #00:05:32-9#

I: Can you tell me about it? The difference #00:05:37-1#

P: First time I was not used to ah the system. It was hard. But now I am used to it now (...) yeah #00:05:44-8#

I: Okay. Ah, what was an especially good and an especially bad interview that you remember? #00:05:51-3#

P: I could say, that it is the first interviews that I have done. #00:05:54-9#

I: Ah okay. #00:05:55-3#

P: These other ones are they are good (inc.). #00:06:01-0#

I: Ahm, what questions do you found, that the people you interviewed ahm fe-felt like hard to answer? #00:06:11-2#

P: You know we do household questionnaires, it is different from women questionnaires, yeah. Women questionnaires are most questions they, when you ask them, they will/ it will be hard for them to answer. Yeah. #00:06:27-1#

I: So we are nearly at the end, so I will just ask you something about your socio-demographic characteristics now. Ahm what ethi/ ethic group do you belong to? #00:06:39-4#
