# Supplementary material for: Gambian cultural beliefs, attitudes and discourse on reproductive health and mortality: Implications for data collection in surveys from the interviewer’s perspective
Source: PLoS One. 2019 May 16;14(5):e0216924. doi: 10.1371/journal.pone.0216924 (PMC6522014; doi:10.1371/journal.pone.0216924)
Supplement: S3 File — (ZIP) [file pone.0216924.s003.zip › S3_interviews/interview_811_0123.pdf]

Interview Four

Setting: Gambakunda, in a courtyard in front of a house of a respondent
Date: 16.03.2016
Time: 14:37
Total interview time: #00:17:58-4#

I: Ahm now I will ask you question about your relationship to the community members, you have been living you have been interviewing. Ahm, how would you describe your relationship with the other mem-members of the community? #00:00:38-9#

P: They are very friendly, kind; nice to work with (.) ja I mean ah (.) we are having a very good relationship with them, yeah. They are very nice and friendly. #00:00:53-8#

I: Ah how did the commun/ How did the community react on your new responsibility, that you had ahm through the interviews you m-made? #00:01:08-0#

P: You mean at the beginning of the interview? #00:01:10-5#

I: Mhm #00:01:11-2#

P: Ahm, yeah the first questions they normally ask, ahm after introducing ourselves, that we are from MRC the HDSS-group, they are normal ask us like, people do normally come, write their name, we tempt to, ahm and lie them more illusions for our coming, like is a different project, I mean (.). We are in the same/ doing the same work, but this time around is (inc.). We do ask questions, cause I mean, about the household thing, and also doing a woman questionnaire (.) that's a women history the the the number of pregnancy, let's see her menstrual cycle, I mean yeah. I think I mean the chosed lady I mean, later to ladies is easier to communicate with your filling lady to, (.) for how to tell you about her miscarriage, her stillbirths, number of abortions (.) yah I mean. I mean talking with them it is very, they they feel very comfortable I mean, I mean yah, I mean (.) before ahm portion in the question and them, we do ask them like it's confidence, what we about to ask them is between (.) you and the person, like is between me and you and (inc.), so with then they feel skilled in. They open up them-themselves. #00:02:31-0#

I: Ahm did your being female had any influences on on the responses from the community? So was it easier for you ah that you are being female to get in contact to the community and ah to ah to do the interviews? #00:02:51-0#

P: Yes yes, because most of my ahm (.) respondents are mainly women, because I am doing women questionnaire. So, most of the time I communicate with only women. So it is very I mean, (.) sometimes it is hectic though (.), but as times goes. I mean (inc.) patient, you have to be patient, tolerant (.) I mean yah, you have to bring yourself down, that all matters in. Yah ah I haven't yet encounter any difficulty, I communicate with them, I get the information that I wanted, (.) yeah with the help of their relatives and (.) yeah I am doing good anyway. #00:03:33-3#

I: Ahm do you feel it is difficult for some women to tell you about their health information? #00:03:42-5#

P: (...) ahm (.) not yet #00:03:45-5#

I: Not yet? #00:03:46-7#

P: Not yet (.) ahm okay maybe they will forget the number of/ the the the time they had the miscarriage, they will remember the number of miscarriage they had, but when you try to tell them like, (.) between which (inc.) you had a miscarriage any, if the miscarriage were not like followed up like, the first time, the second miscarriage (.) if they have to start from like my my second was a miscarriage, them my second was/ had miscarriage, it is bit confusion, (.) I mean you have to note it down, then like the you will be starting from one, like you will be saying to the person, that you say it like, "your first birth was a live birth, or a miscarriage or a stillbirth " (.) then even though it was/ that was right, they will say you "no, it was a live birth". So and that is the kind of difficulties we have, like if the person have ahm (.) quite number of miscarriage or stillbirth, yeah. But when we come to live births and (.) yah it is very easy. #00:04:43-5#

I: Are there certain people who find it more difficult to answer some questions, like elderly women or young women, some people from ethic groups, do you have differences there? #00:04:55-3#

P: Ahm yes, when you come to like elderly women women, like maybe they would consider your age, and you asking them about your/ their menstruation cycle I mean, they feel a bit sad, or they feel a bit discriminated, like they try so (inc.) so one that you can born like (inc.) asking that kind of questions, like life around is very (inc.) sometimes very difficult, but you have to make them understand. (.) like, just I said earlier be down to ask, I mean (.) just takes a while (.) it is/ while coming, it is about health and the information that you are getting from them is very important. (.) So sometimes you encounter such a a a a difficulty, but at the end, we don't force them, ya it is just deal with patient, at the end they will tell "okay" they will start giving you the quest/ answers that you need, (.) you want from them. #00:05:56-1#

I: Now, we will come to the general work ah fieldwork experiences. Ahm, please tell me about the experiences during the fieldwork #00:06:05-8#

P: Mh (...) ahm, it was a really nice experience, I have never been fieldwork-work before, I haven't done, meeting with different kind of people (.) it's I mean, going to so many places, I mean, I have been to so many places that I never known and like (inc.) (.) in The Gambia, I mean, Gam/ people will say that Gambia is too small, but I have never visit to so different areas, yeah, I have never imagine that there are so many places are in The Gambia here. (.) And the people, like I said, they are very warm and friendly, they they are good. (.) Yeah, I have a very good experience though. (.) Yeah #00:06:45-9#

I: Ahm, what do you think went well? #00:06:49-4#

P: (...) mh, (.) what I think went well? #00:06:54-7#

I: mhm #00:06:55-2#

P: I think for now everything is going well, we getting information from them (.), ahm they are opening up themselves (...). Yah (.), the work us for now it's going very smart experience, for me and I guess with all cp-workers also (.), it's going on. We're getting all the information that we need. #00:07:17-8#

I: What were the challenges? #00:07:20-8#

P: Ah, the challenge ah sometimes (.) when you come to a women questionnaire, maybe you find the person sleeping (.), ahm they have to wake the person up (.) yeah (...) yeah she won't have the time, s-so you have to/ the person have to ask you to wait for (.) her, or to go and come some other time (.) yeah and also to have (.) papers from them sometimes is very difficult (.) yeah, like when you ask them, how many passes she have (.) yeah, if she already gave you (.) the number of birth she have, like to get the passes of the kid is difficult. And when sometimes, when you try to come from, like you don't know, like the person says he hadn't had the passes with, so you're trying to ask like "how old do you think your child is?" (.) sometimes the t/ the question the answer they will tell you will be "I don't know" (.) so (.) that kind of/ it's it's really hectic, because you do have/ Normal what we always do is to prom, (.) yeah at least to know the number of they have, the the age of the last birth, like if he is like five or six years old, then you ask her like "how many years interval you will think is between the fi/last and the second last?" Yeah then that's how you do, you try really go, and that's how we normal do it. But the paperwork is the only difficulty, or sometimes go to a certain compound and you will find no one there, they are all out, like they go to the farm (.),or they go to (inc.) market (.) yeah, th-that are the/ or sometimes (.) the the women ah questionnaire, the household questionnaire you will do the interview, they will stay you waiting and they will write a particularly/ she will write a particular ahm women as (inc.) but you cannot/ you doing the women questionnaire you cannot interview her, because she is no way to be found, maybe they say she go to the farm, some places they will maybe tell you they go 8 in the morning and they come back 8 in the evening (.) so normally it's very difficult, but that's rare it's not (.) (inc. unclearly spoken) #00:09:35-2#

I: Ahm do you have/ Oh no. Do you have any positive experiences? #00:09:41-5#

P: Yes, (.) @(.)@ ya yes (.) ahm (.) ya, I have really good experience I mean, (...) ya like, (.) just as I said ahm (.), talking to them I mean, sometimes people won't (.) like to tell you their experience, like having miscarriages, is it's sad to lose your child, I mean to have a stillbirth, but sometimes ahm, through my review some people they feel like happy to to say it (.). like they feel comfortable after saying it, they feel like their released (.), like there maybe have been years and years, they didn't talk about their pregnancy (.), so you come in to them, talking with them I mean (.) with a (...), a very clean head ((or heard)), like be (inc. unclearly spoken) I mean (.) Yah it's a positive thing (.) yeah #00:10:40-0#

I: Did you have any negative experiences? #00:10:43-5#

P: (...) in this fieldwork? (...) Just as I said, the the (inc. , unclearly spoken) not found at all (inc., unclearly spoken). That is the only negative thing I experienced. #00:11:01-4#

I: Do you have a s-suggestion how this could be solved? #00:11:05-3#

P: Mh (.) well, I don't know. Ahm the only thing that I think would be possible way to go with

them at their workplace. But that would be if they allow it. #00:11:20-7#

I: mhm #00:11:21-1#

P: Ya, I mean maybe if we go to their workplace you will find them busy, so if they don't have time for you to be interviewed. Ahm so normally, what I do suggestion myself sometimes is like, if we can ask, like their neighbors, like if we don't find them, sometimes we do find some like the husband, (inc.) the women goes to the f/ ah the garden, yeah maybe you will ask the husband "can I come the other day, can I come maybe tomorrow" or "if we come will she allow me to interview her on her field?"(.) yeah, but that if the women are settle #00:11:58-1#

I: Okay, can you remember the first and the last interview that you made? #00:12:03-4#

P: Yes //@(.)@@// #00:12:05-8#

I: //@(.)@// Can you tell me about it? And the difference between them? #00:12:08-6#

P: Okay, the the first I have was during the training (.) yeah I going to a house were they give me a household, I went there (.), the the person they are moved, they said he moved. It was no way to be found, so (.) I called my coordinator like to ask (.) "What should I do?" So he told me "If there is some other person, in the household, you interview the person" (.) so I went to that person (.), he said, he didn't have the aim, he refused to be interviewed, so I called back again, he told me "Okay, (.) take the next door, the next name (inc., unclearly spoken)" so, I did the interview the person, it was (.) tuff? Yeah (.), because the women was spoken here and they are working, as I asked her like if she didn't had the time, that I can come tomorrow time. She told me "no", we can visit her, so she was doing the work, at the same time responding to my questions. So it take a little bit time, (.) it take a l-lot of time. (.) Yeah, that was/ but I have all the information (inc.), the time I spend there was so nice (.) yeah. Ahm the last interview that I did, was yesterday. (.) Yah it was (.) very small. I went there, I f/ I found everyone there, all the women at their men, and they brought all the children’s documents for me,(.) and it was (.) yeah I had a very nice interview. Yeah we spend like nothing less than one hour in the household. #00:13:36-1#

I: Mhm #00:13:36-2#

P: (.) With three, with four to five women questionnaires, so I think that's/ there was a huge difference in that. At the beginning it was a starting women, it was during the training, so I I mind worst, like when asking the question I do take my time, ask them as time goes, you get used to it, (.) yeah (.) so (.) yeah, it's just like they do their thing, (.) it was a really good experience, yeah. #00:14:06-2#

I: Ahm, was an especially good and a especially bad interview that you ah performed? And ah were was the difference? #00:14:16-9#

P: The best? #00:14:18-3#

I: A a really good interview and a really bad interview? #00:14:23-3#

P: Mh (...) yeah, the bad interview I have was, (.) the first time we went to Numiel, yeah (.) that was the bad, I mean (.) yeah I interviewed a women, till we were about to go to the siblings, (.) I asked her the number of births that the mother give to (.), she refused the. (.) So she told me okay, "there are I can't say the exactly number" so I told her like, how many people are older than you?" She told me like (.), there are five (inc.) and I think she was the last born, six or yah six, she was the last one, so I ask her to give me the name, she refused, so (.) unless you did an interview this bad thing (inc. unclearly spoken). I leave the space black, because the name were/ she refused to give me their name. Yeah the best ahm (.) interview I have (.) I think it was today (.), only as I said, all the papers were brought and I found everyone of them there, and they were very nice and (.) we have a very comfo-fortable to to stay there, though we normally have a very comfortable place to stay, but this one it was different, because (.) it was just like looking at the paper and writing it down, because ah yah, it was very good. #00:15:53-9#

I: Ahm, what were the questions you find most difficult to ask in the interview? #00:16:02-0#

P: (...) Mh. (.) Menstruation cycle, (.) yeah because some women, they feel shy telling me that and also were ever the person is pregnant (.), sometimes they find it difficult, the person is pregnant (inc.) you ask her about her menstruation cycle, she said "I haven't seen it for months". And you've ask the person if she is pregnant, even though she is, she will tell you "I am not" (.) Yeah, is (.) sometimes you had to encounter them. (.) Yeah I have encountered that and I have wrote it down, like (.) I told the person, like "Are you not sure or are you not?" , she said " Nothing is wrong with her, she is not pregnant" so after the mother have to tell me that, when she was away the mother told me that she is lying, she is pregnant, she is three months pregnant. I have to go back, and added that she is pregnant. #00:17:01-8#

I: Ahm, what questions to you feel ahm the respondents found hard to answer? #00:17:10-7#

P: If she had a (inc.) is pregnant, to ask wherever they are pregnant. #00:17:18-1#

I: Ahm so now we are nearly at the end, I will just ask you a few questions about you. Ah, which ethic group do you belong to? #00:17:27-7#

I: Do you want to add anything at the end? #00:17:46-4#

P: Yeah, I am very happy with this interview, thank you so much (.) yeah. At least I got to someone my experiences in the work and (.) it's it it was something great to me. Thank you very much. #00:17:58-4#
