# Supplementary material for: Gambian cultural beliefs, attitudes and discourse on reproductive health and mortality: Implications for data collection in surveys from the interviewer’s perspective
Source: PLoS One. 2019 May 16;14(5):e0216924. doi: 10.1371/journal.pone.0216924 (PMC6522014; doi:10.1371/journal.pone.0216924)
Supplement: S3 File — (ZIP) [file pone.0216924.s003.zip › S3_interviews/interview_811_0124.pdf]

Interview Five

Setting: Gambakunda, in a courtyard in front of a house of a respondent
Date: 16.03.2016
Time: 14:58
Total interview time:

I: Ah okay (...) Ahm so now I will ask you some questions about your relationship with the community members.

P: It really is very perfect, with me, because like if we are working with the community, you shouldn't have the trouble, because when you go to the people, you always have the social things, like you go to them, you eat what they eat, like you feel how they feel and at the end you will get what you need. Yes.

I: So, you have a good relationship with the community members?

P: Our relationship is very "conidial" because, like if you are in the community you didn't know what being once there, like finish survey, you are paid to do a survey, but the member, the respondents, they are not paid to respond to your questions, so you did it you did it used to have the relationship and get what you need at the end of the day. But if the relationship, the quality of relationship is not perfect, at the end of the day you dont get what you need.

I:Yah

P: Yes

I: Ahm, how did the community react to you and your responsibility with the interviews?

P: Okay, though sometimes is hard, because some people they have bad mind against the MRC. Because some people, if you tell them MRC, they have bad philosophy (inc.), but they say MRC they normally used to take some people bleeding, bloods, and also those others like wherever you see a advantage, at this advantage (inc.) is there. Other people also they didn't (.) ah like MRC's activity, because they know they say (inc.) whenever they are sick, MRC used to take good care of those and so they refer they they refer being they are very happy of that so when you go to those co/ ques/ ah compounds you really met that, like their family, is not informed. Like you the interviewer, or you the person who is there, you should have the motivation skills, not like giving them money, but whats. mhm (inc., unclearly spoken) convince them, even they are not will to answer to your question and at the end of the day you will have that (inc.) they answer to your questions. (.) Yes.

I: Ahm, what is your impression?

P: Mh?

I: What is your impression of the community?

P: I did like them much, because the family we are are that day before yesterday, we were at Dembakunda, but we didn't used to this household, we didn't like the house/ family, we treated them nice, we went to the Bantaba, we had a seat at then all man called us that comfort lounge, (inc., unclearly spoken) you know is where 27 of or 21 of us where there, came in for. Just remember just remember 21, she catered everything, like yesterday too (inc., unclearly spoken) you even interviewed her compound, so we didn't know even when her household is among, of those number households we should interview, than today she is part of the interview (inc.). (.) So, you know the community, though the really been good. They are perfect, even sometimes some people don't give you breakfast, they don't care for you, but when you are in their compound, they get you breakfast, they will do everything for you. And also what I told you hat the beginning, remember for once we are paid to do interview, but for them, they are not paid for respond our questions, so you have to make easy easy and at the end of the day you will get you wish from them

I: Did your being female had any influence on the responses ahm from the community? So did they react like different, because you are a female, did you had that ex/ impression?

P: Nonononononono, here like we are females, (inc.) they didn't act like what/ when we are doing the interaction, you know the HDSS team they are both males and female. But during this survey, we are all ladies, due to the the questions, we are asking. So hadn't been/ I I know it hadn't been (inc. (towards)) men involved, it would not be suitable. (.) yes

I: Ahm, it is difficult for some women to ah tell you about the health information?

P: Here no, women always feel free, like when we were saying, even the ethic of m/ your parents, the will tell you (inc.), like is nothing here, but is heart wise, you will a mother say "don't worry", just them how you feel, like if you ask the question, (inc.) "I'am sorry, really sorry", I said "Hey, nothing that I don't feel, I don't think bad of you. It's fine (inc.)" So, we didn't had any difficulties about that (inc.).

I: Okay, ahm so now we will come to your fieldwork experiences generally. Ah, please tell me about your experiences during the fieldwork.

P: (inc.) Though, me, before MRC I was working in the field before, because I was working the ah first time for three years in the field, so community (.) is so, is not my problem, like because all ah I know one thing, like if you are in the, if you go to the fieldwork, you know how they they f/ live with tho/ live with them and you see many of their cultures, you respect them (inc.), and also if they offer you (inc., unclearly spoken) even though you are in personal effect, but TAKE IT, let them know (.) you feel how they feel. Mh and you better respect them (inc.). What is true between you and them, leave them as respected. Yes, those people here, (inc.) they don't aspect (inc. unclearly spoken) and I do like them for that, yes. They give you whole heart out here

I:Yes

P: And at the end of the day they expecting nothing from you, (.) yes.

I:Ah what do you think went well?

P: Mh?

I: What was good?

P: What was good? During the interview?

I: Interviews and your fieldwork experiences

P: This were good with us, like we don't have any difficulties, ME my team

I: Mhm

P: I cannot spoke on we had ever one, like my team during that fieldwork, we don't have any difficulties, like when we are giving the number of households, we normal do that, aspect that, like when we miss some people ah, my friends they went out from the (.) village we will try to replace the other household and when we replace the other household, they are really proud and happy for them to receive us nicely and we do our job expected (.) yes.

I: Ahm what were challenges for you?

P: (.) During the field or?

I: Mhm

P: During the field here. (.) Though wherever you see advantages, there is a disadvantage, like the issue there is (.), we are doing as expected mhm, do to do this work as (inc.) we ask for mh, because when/ and also when we are doing things we don't know what will be as/ what (inc.) is expected from you, as you are there. But the challenges we have is like, when we pay/ when we are paid mh, it will be very very difficult to ask them, because if you know like you look like your (inc.) and you are working for better money, to like your family what they are expecting from you at the end of the day mh, it is when they are expected from you. Sometimes it's you money before that end of the payments, (.) yes. And that is (inc.) telling our difficulties, like we all/ the money we are having is not enough for us. (.) As if we look at like (.) look at this (inc.), mhm, from morning to for o'clock (.) go home. Yeah, working for the comp/compound, moving from one place to another. yes.

I: Did you have any positive experiences?

P: Yes, I have worked with MRC before, but the I know also, you know when working with MRC you always have the correct manner, and also if you do better you even though what you are doing any fieldwork you will you will be ready to work with them (inc.). I am really happy, working with them, but also I have gained a lot of experiences in the field. (...) And now it improved my standard.

I: Did you have any negative experiences?

P:((laughing)) Negative experience like how?

I: Ahm (.) generally, what was negative about about fieldwork? Or, was everything fine? Your

staying? Your your accommodation? //And//

P: //Yes// yes like the time we were in Bakaday, (.) is there was a negative experience we had, like the mattresses they bought for us, it was not up to standard (.). Like some people their health conditions, won't allow them to stay. (.) And sometimes also our feeding is not up to standard, like (.) if we are said/ we are feeding we we are feeding ourselves, like the morning we are having is all going for our feeding. (.) (inc.) If you are being/ if you are eating every day 200 Dalasis and you are paid 200 Dalasi per day, than what are you keeping at the end of the day?

I: mhm

P: So, that was our problem, as I told you before, like the finance was our problem. And the standard of the mattresses, very bad.

I: Ahm can you the first and the last interview, that you performed?

P: My first interview I performed? Yes, (.) the time I was/ the time we were on our training

I: Mhm

P: That time, that we are pretesting (inc., unclearly spoken). So, the first interview, I went to a compound of [...] mhm, but the wifes name is [...]. So (inc.) came out when they asked for the husband, she told me that (.), the husband is/ I asked her about her husband and said "Where/ Who is the wife?" she said "Or MRC (.) fieldworkers who normally used to come to the compound, they always ask/ they always know that compounds are their/ compounds first name and the wifes name and the kids". (.) Yes (.) so, I didn't f/ I was even scared so I went and called the fieldworker (inc.) he told me the older women and the kids so I came back to the compound and I "Is is okay? Perfect!" Then she allowed me to do the interview. Yes (...)

I: //And the//

P://And the last interview ((laughing)) we are we are, it was very very perfect, though people were (.) much in the compound, because there were 79 (.) in the compound. But the grandpa was willing to listed everybody in the compound as expected and give us all the details we needed and all the women were pleased and we (inc.). (.)Yes.

I: Okay, so ahm what was an especially good and an especially bad interview for your/ for you?

P: Good and bad?

I: Yeah

P: I am really proud of all the interview I did (.). Yes, even if Dr. Anne walk to the interview, me and my team did, we don't (inc.) information, we we don't influence her, because this is a a research (inc. unclearly spoken). Always, if you are in the field, do as aspected of you (.) yes, me and my team, we don't force data, (...) we don't, even like do my report, if I don't

get ah (.) the whole data, that I needed I will just say, pending data, so if I am giving time, I will (inc.) back. (.) Yes. I don't want to force data. I don't want to be blamed. (.) Yes.

I: What were the questions you found most difficult to ask?

P: (...)(inc. ,unclearly spoken) sometimes is difficult, because you ask about the data (.) (inc., unclearly spoken) their deliveries or their births, but though the women questionnaire sometimes like (inc.), they have language barrier in this community, but me I can try to (inc.) little, like they can ask about their menstrual aspect of the women so I, so as I tell you before starting the interview I did tell them that sorry, "sorry I know you mind but sorry is health" so I interviewed, after that (inc.) the interview to, I apologies. So everything is fine (.) yes.

I: Ah what do you feel the respondents, so the people you were ah interviewing found hard to answer?

P: Serious, the question that we have is not much hard like ap/ ah (.) if you look at the women questionnaire (.) yes. The the women questionnaire what is difficult is like the menstrual aspect. Yes (.) miscarriage, when you ask someone about the miscarriage, it is very a big deal. (.) Yes, but like when asking the sister, another sister or aunty or stepmom or anyone about the menstrual aspect, is not easy you know, but it's fine, is your job, (.) yes.

I:So we are nearly at the end I will just ask you some questions about you. Ahm which ethic group do you belong to?

I: Do you want to add anything at the end?

P: At the end, I am really pleased (.) yes, thank you very very much for the interview, I am really pleased, and also if you asked me if you are comfortable, if the questions are really answered as expected, if they are not answered as expected, we can still go for go for the interview. (.) I have much time, though I guess I should enter a few things in my tablets, like for preparence for tomorrow, but no problem, I should as if you are comfortable and satisfied. (.) Yes.
