# Supplementary material for: Gambian cultural beliefs, attitudes and discourse on reproductive health and mortality: Implications for data collection in surveys from the interviewer’s perspective
Source: PLoS One. 2019 May 16;14(5):e0216924. doi: 10.1371/journal.pone.0216924 (PMC6522014; doi:10.1371/journal.pone.0216924)
Supplement: S3 File — (ZIP) [file pone.0216924.s003.zip › S3_interviews/interview_811_0125 & 811_0126.pdf]

Interview Six

Setting: Gambakunda, in a courtyard in front of a house of a respondent
Date: 16.03.2016
Time: 15:14
Total interview time: First file (811_0125): ,
Second file (811_0126):

I: Ahm, now we will come to th/ your releationship with the community members.

P: Mh okay

I: How would you describe you relationship with the other com/ members of the community?

P: Ahm, (.) sometimes it's not easy and (.) certain communities, if you go there you have the fear, that they might reject you or they might even (.) put you in a situation, that you can not even can introduce yourself. But some (.) stiua/ some situations it is easy, if you go some of them they welcome you, but most of the cases you have the fear, (.) you have the fear that, ah they might reject me or they might not welcome me. Some they will welcome you, some they can't welcome you. So it depends,(.) how the situation of the society is.

I: Okay

P: Yah

I: Ah, is it a good relationship to the community, (.) that you have?

P: Yeah (.), I mostly I always have good f/ ah good relation with them, because sometimes I have a quite good relationship with them, sometimes before even having my work started, sometimes I do chat with them, at the front, then I continue the job. Some, before I even start, I told them "(inc.) This is my name my, this is my surname" I ask their surname, them (.) I start a (inc.) joke for a while, then I continue with the job. (.) Yeah

I: Okay, how did the community react on your new responsibility?

P: Yeah, sometimes they aprove it very wellcomely (.), but some of the situations (.) it will not be easy. Some of them they reflact faces, so you are in fear ah their relationship with you (.) will be harsh. But thank god that some of them/ (.), even though, but as time goes and you are introducing yourself, they give you the free chance and they will welcome you warmly. Some of them will even go ahead to give you lunch (.), some will give you many gifts. (.) Yeah

I: Ah, what is you impression?

P: Yah, my impression is that, I have a quite lot of knowledge, I did a good adventure at is and the other thing is that, (.) I learn a lot and I learn how to approach people, how to go

with people and how to move with (.), even my colleagues at work. So I learn a lot about it. (.) Yeah.

I: Did your being female have any influences on the re-respones from the community?

P: Yeah (.), they do. Some of them, if you ask them, they will feel (.) ah you are offending them. Because some of them, they will have it that they, if can remind them at that time of the death, it will be really ta/ it will be really hard on them. (.) //mhm// So it will take time. It depends when you, how you will act with them, so it will take time (.), to give them a little time, if you ask them question, you feel simpathy with them. (.) //mhm// Some females, they will feel very very (.) or/ a/ they'll feel very disturbed, if you ask them certain things like that.

I: Ahm, do you feel it is difficult for some women to tell you about their health information?

P: Yeah, some of them, like me, if I go to a place there was a lady I wanted to interview, I find it very difficult. Because she was not willing to give me the information, and (.) she was not (inc.), she was not having any concern about it. And that when it was very hard to do that one.

I: Ahm are there certain people who find it more difficult? Like eldery women, younger women to answer (.) ahm questions?

P: Yeah, some womens, they have it difficult, because, if you ask them some questions, they will feel like "Ah, this question is in my private, so I don't need to share it with anyone. And private is private." So it's very hard on t/ on sometimes, like this menstrual period, if you ask them about their menstrual period thing, the will not even bother to tell you "I don't know". (.) As a direkt answer "I don't know" (.), so it's very hard. Mhm

I: So, now we'll come to your general work experiences, fieldwork experieneces.

P: Mhm

I: Ah, please tell me about your experiences during the fieldwork.

P: @(.)@ The field, it's very hard. (.) It's not easy at all, like we stayed to a village, just lie down was the problem. (.) Yeah, because the matresses were very small, (.) I had a (inc.) difficulty, my chest do pain a little while, because of the due to the condition, we have to go to a pumping mashine, to pump your water (.) at the morning, and then we find breakfirst and others stuffs. Sometimes to get to a village, it will be difficult. Some of the ways are not good to an extern, that you can go easyly, (.) going there it's very very hard. Espacially a place "Dambalanga", it was very far and ver/ very difficult to go there. The other thing is that, the people you will find too, it will be very hard, to have their attention to you, it will be very hard, because they don't know us, we don't know them, that's the first time and that's everything that begins on that time. So it will not be easy at all. (.) The field acutally it's not easy, it's not easy at all. (.) Yeah.

I: What do you think went well?

P: Mh?

I: What do you think went well? What was good?

P: Yeah, what was good is that, when ever a information complete, that was very great. And the other thing is, (.) if you find it very easy (.), very easy on going, coming, that was very good. Especially the information. The information, that's the best thing. If you have all the information, that is good. But if you don't have it, you feel bad and you feel disapointed. //mhm// Yeah

I: Ah, what were the challanges?

P: Yeah, the challanges is (.) sometimes, we have to go to a compound, they (inc.) you. Sometimes you'll have to go to a certain village, before you'll find a compound, it will take you almoust two hours, three hours, because some of the numbers. Or you go to a place, like here, yesterday I came here, to find the other two compounds, I find it today, (.) because similar names are occuring, (.) nicknames, their their the AKA. The AKA too, they are sometimes they are all similar, so to find those things out, it will not be easy. Sometimes you go to a compound, he will ask you "What is the first name of the son, or the daughter the name they give you", so we will tell them "Well, we are new, we don't know them and unfortunatly we cannot tell, who and who is under him or her" (.). That thing, it's not easy.

I: Did you have any positive experiences?

P: Yeah, I have a positive experience. (.) The positive experience is, how to live (.) apart from my home, how to live outside, how to live with villages and how to live without my family. (.) And then how to live on my own, (.) because I have never been alone for a long, without my fa family, so now I am fond of it, and I would really aprreciated it. Yeah.

I: What/ ah, did you have any negative experiences?

P: Yeah, the negative experience is like ah (.) sometimes, to go to a village I will find my little pain, I'll sometimes go to the field, if I should sit down, without any information complete, (.) ah that thing is very very. And the neg/ the the other negative thing is that, some of the villages, (.) the language barrier is there. (.) //mhm// Yeah. The language barrier is very very difficult. Like me I can try Mandinka a little bit. I am a Fula. So Sarahule I can't. The other languages I can't. Wolof I can. But the others apart from Fula, English, Wolof, the others I can only try. (.) Yeah.

I: Do you have any suggestion how this ahm could be solved?

P: Yeah, like staying at the villages, (.) for instance (.) if, we want to work at the villages, some of the villages the distance (.), it's very difficult. And sometimes we will find it very hard for one vehicle to take us. Sometimes we will find it hard for food, sometimes we find it hard to even our clothing ourselves, sometimes we have to keep our dirty clothes aside, if you have a weekend (inc.), like normal suggestion is in this, if only there is a solution to this, if they can provide two vehicles at the field at one time and then the food. When you are staying at the villages, without any food on you, you can not work. You can not even control yourself, talkless of working. So, if they can provide the food and the accomondation that is

very comfortable. Because sometimes, if you go to a (.) village expert like here, you don't had a house, we had to stay comfortable, you can not do your work at the time. //mhm//So if you have a comfortable place and a good food provison, you can maybe (.) yeah. That things at least, it can improve your intern and extern (inc.), you can do, even (.) even if you are feeling tierd, but you have a little rest, you can continue your work willingly. You can do it willingly and without even having to mind, that ah if I go home, I will have to find food, I have to find were to sleep, I have to find my shop my everything. So if they can provide all those things, (.) it can be (.) it can be little bit okay. //mhm// (.) yeah.

I: Can you remember the first and the last interview, that you performed?

P: Yah, the first interview I did I was shaking, I was feeling that "wow, if I ask about it", like this question "Did you have any deaths that occured during this year?", wow, they might even hit me, because I was feeling scared, because I know how it feels to remind someone that, who passed away and who even (inc.) about age (inc.) abortion

I: I only stop for a sec.

>> Video stopped and continued with file 811_0126<<

I: So we will continue, (...) so the first and the last interview

P: Yeah, the first day it was terryfiing, it was very very difficult, because I went to a society that I have never known them, they have never know me and then the other thing is "Who is this guy, who is she to interview me, how is she coming to my compound just like that to tell me all those questions". (.) But as time goes on, my last day I am fond of it, I can enter to a compound and chat, do many things before I even interview. Sometimes I (.) try a joke between (inc.) relationship, (inc.) and etc. . So I'll tell them that "Hey your igdoma" or you do this, to have their attention to me, so (.) that is how it is. Now the last day it's okay, but the first day it was very very difficult //for me//.

I: //Yeah//

I: Ahm, what was an espacially good and an espaccially bad interview? And where was the differnce?

P: Sorry?

I: An espaciall/ (..., really loud background noises) @(.)@ An espacilly good and a espacially bad interview?

P: The bad interview is, you will go to a compound (...) you will go to a compound, without them paying attention to you (.). They will leave you, because some of them, they will ignore you for something. I go to a compound, they ignored me for almoust an hour. (.) I was just being there, trying to have their attention to me, but thank god that they lastly have their attention to me. The other thing is that, I went to a compound, they were very welcoming (.), the welcomed me without even me knowing them, they don't know me, they don't know even where I am from. //mhm// So they welcomed me, but the others, it is very hard. You can (.) you can not just come and have their attention like that. It will take time. There was a

lady too, before I even say "Hi", she rejected me, instandly. And the next move I keept patient, she kept me for the hot sun for a while, (.) I asked her still, wherever she can give me the time, she said "Okay" she can, but immedientily I interviewed her before I sit down, she said I am done. So it was very hard. But the others, it's easy. (.) Yeah

I: Ahm,(.) what were the questions you found most difficult to ask?

P: The question found most difficult to ask, is wherever your parents alived. //mhm// (.) Sometimes some of them, their parents are not alive. But to ask them that, it's not easy. Some to ask them "How many deaths occured during the last year" Some they will tell you "Acutally there is" (.) you see them feeling, something else. So you have to feel with them //mhm// if not, they cannot they cannot cope with you, because you ask them something that is very difficult to remember, they are love ones, they lost them (.), so to remember those ones and tell you about them (.) it is not easy. They they most have something they feel in them. (.) So that question (.), I find it hard. Sometimes I tempt to keep it, for a while, (.) at the end of the interview, I tempt to ask them, because I have the information apart from that one. So, if I first beginn with that one, the interview might close. So that question (.), is little bit bothering to me. //mhm// Yeah (.), because if somebody would ask me about the deaths ones that I have, I will feel something, that is why that question is something difficult for me.

I: What were/ what was the questions you feel the respondents, you were interviewing, found hard to answer?

P: Yeah, she we find it hard to answer, espacially if she delivered, she is divorced with the husband and I ask her "Who is the owner of the compound?" //mhm// If it was the husband, sometimes/ they know what is going between them, but some of them they will tempt to say " I am the one". Some of them, they will not tell you (.), some they will not ne hesitatet do tell you " my husband is the one", but some (.) you cannot find it easy on them. It will be depending on how their situation is, but to ask them those questions, it will be not easy. And the women (inc.) you go there, the husband has tell you. You ask "Who is the owner of the compound?", they might tell you "well, is my son" (.) now before it is the dad, to tell you it was the dad and still it is the dad, they didn't changed it. It will be difficult, they will like giving with you, exept if you just make it clear to them, it is the one that still was the compound, they did change it. That want one too is there.

I: So we are nearly at the end, I will just ask you some last questions about your so-socio-demograph/ demograpic characteristics.

I: Do you want ah, anything to add at the end?

P: Yeah, just to say, (.) may it be in a peaceful contract, (.) and may it continue in a way that everybody will be pleased and at the end to say a goodbye in a beautiful face, without (inc.) or saying hat I known. You will never have that experience (.) yeah. Thank you!
