# Supplementary material for: Gambian cultural beliefs, attitudes and discourse on reproductive health and mortality: Implications for data collection in surveys from the interviewer’s perspective
Source: PLoS One. 2019 May 16;14(5):e0216924. doi: 10.1371/journal.pone.0216924 (PMC6522014; doi:10.1371/journal.pone.0216924)
Supplement: S3 File — (ZIP) [file pone.0216924.s003.zip › S3_interviews/interview_811_0127.pdf]

Interview seven

Setting: Gambakunda, in a courtyard in front of a house of a respondent
Date: 16.03.2016
Time: 15:32
Total interview time: #00:08:06-4#

I: Okay, so now I'll ask you some questions about the your relationship with the community members. How would you describe your relation with the other members of the community? #00:00:39-2#

P: Mhm, what do you mean, all the members or? #00:00:41-5#

I: Yeah, you have been in the fieldwork with. The community #00:00:47-0#

P: I I don't understand. #00:00:51-0#

I: Like, ahm how would you describe your relationship with the community? While while you are doing the //fieldwork//. #00:00:58-8#

P: //Ah is// good. #00:01:00-0#

I: Yeah? #00:01:00-6#

P: Oh yeah #00:01:02-3#

I: Ahm how did the community react on your new responsibility? So that you were doing the interviews with them. #00:01:09-8#

P: The interviews. #00:01:10-4#

I: Yeah #00:01:10-7#

P: Some do really appreciate it, but some are just not appreciating //mhm//. They don't appreciate it at all. Yeah, some they will send you away, because they don't want the interview. #00:01:20-1#

I: Yeah. (...) Ahm, what is your impression in general? #00:01:25-4#

P: Like? #00:01:27-0#

I: Ahm (...), yeah ahm about your work with the community? Was is good, was it bad? #00:01:36-1#

P: Was very well. //mhm// Okay it's good at times interviewing, some are very welcoming. You ask them questions, they answer you, they give you the answers that you want. And they give you/ but some you question them, they refuse to answer, or they will refuse to give you the answers that you need. //mhm// (.) Yes, or you start interviewing, you go up to the middle, then they tell you, they are tired of the interview, they cannot continue the interview //mhm//. #00:02:01-3#

I: Ah did your being a female have any in influence on the re-re-responses, so the people you have been interviewing, ah from the community? So did that like make a difference, that you are being female // to the community? // #00:02:21-5#

P: //Yeah// (.) because I am learning from them too. //mhm// Yeah #00:02:23-1#

I: Ahm do you feel it is difficult for some women to tell you about their health information? #00:02:30-6#

P: Yeah, it's very difficult for some //mhm//. Yeah. Especially, when they have this miscarriages or abortions, it's very difficult. //mhm// Yeah. #00:02:40-3#

I: Why do you think it is difficult for them? #00:02:43-0#

P: Yeah, because it is their personal life, that's why, they don't want to discuss it. //mhm// #00:02:48-1#

I: Ahm are there certain people who find it more difficult like eldery women, young women, people from a certain ethic group, to answer those questions? #00:02:59-9#

P: Ah no, can you repeat this //question//? #00:03:03-6#

I: Yah, are there certain people who find it more difficult that others, //to// /? #00:03:09-0#

P: //Yeah// #00:03:09-0#

I: Yeah? (.) Which ah, which especially, what do you //mean//? #00:03:13-6#

P: Like 15, 18. //mhm// Yeah 15 to 18 years. So we ask them that, "I am not married, so I don't have this experiences, I don't have anything to answer." #00:03:23-6#

I: Ah okay. (.) So now we will come to your general fieldwork experiences. Ahm, please tell me about the experiences in the fieldwork. #00:03:34-2#

P: My experiences is very good, because I have learned how to socialize with people, how to be with different different people, have seen different people, different attitudes and like I have received different answers to from them. //mhm// (.) I have learned from them too. (.) Yeah. #00:03:49-5#

I: Ah, what do you think went well? So what was good? #00:03:54-6#

P: What was good? Socializing with them, yeah. And meeting new people, making new friends. (.) Yeah #00:04:02-1#

I: What were the challenges? #00:04:03-6#

P: The challenges, (.) the challenges were like it's so difficult in some of the a-areas. Food is

problem in some of the areas, the food we have done in Basse, what we are used to eat, is not the same //mhm// for that day eating. The water is the same. (.) Yeah. #00:04:20-8#

I: Did you have any positive experiences? #00:04:23-5#

P: Any positive experiences? (...) Yeah. #00:04:28-9#

I: Do you want to talk about them? #00:04:31-3#

P:@(.)@ no. #00:04:31-9#

I: Ahm, did you have any negative experiences? #00:04:36-3#

P: Yes #00:04:37-0#

I: Do you want to describe them further? #00:04:40-7#

P: Like the negative experiences I had? #00:04:44-7#

I: Mhm (agreeing) #00:04:44-4#

P: Yeah, just like I told you before. Some are not just welcoming, you will just enter, you start explaining yourself, they will not even allow you to finish and they will send you away. //mhm// (.) Yeah. #00:04:54-9#

I: Do you have a suggestion, so an idea, ah about how this could be solved? #00:05:00-6#

P: No, I don't thinks so. #00:05:03-3#

I: Ah, can you remember the first and the last interview you performed? #00:05:08-7#

P: The first and the last? Is it the place or the women? #00:05:12-5#

I: Ah, the interview. #00:05:13-7#

P: The interview. #00:05:14-6#

I: Yeah # #00:05:15-0#

P: Well, it was at Basse //mhm// (.) Yeah. (...) but I can't remember the name of //the women. Yeah the last I did//. #00:05:26-5#

I: //No you don't, just the general experiences// #00:05:26-5#

P: was [...] //mhm// in (...) how is it called? Dembakundakrumba, [...] was the last women I interviewed. #00:05:34-5#

I: And was there a difference, ah between the interviews? Like you were performing or ahm the people who were interviewed were performing? Do you remember that? #00:05:44-5#

P: Performing like? #00:05:45-8#

I: You performing the interview, was there a difference //between the first// / #00:05:49-7#

P: // Yah//, between the first and the last, yes. (.) Because at first, myself I @(.)@ was, I was no and I didn't have the experience, so it was very hard for me. //mhm// But for now I am used to it. #00:06:00-3#

I: Ahm can you describe your different experiences between the interviews? #00:06:05-6#

P: Between the interviews? //mhm// (agreeing) Like the different I had? (.) Like you know, with different peoples it's just so different, because you meet this person, so she will be willing to answer that you need from her. But at times you meet this other person, she will be not ready to give you all you need from her. (.) Yeah #00:06:23-7#

I: Ahm can you remember one especially good and one especially bad interview, that you experienced? #00:06:32-4#

P: That I experienced? //mhm// Yes, (.) one ah the lady called [...] in Basse, no one was @(.)@ so bad. #00:06:41-9#

I: Okay //Why?// #00:06:43-0#

P: //Yeah// .Ahm, because the lady, I started interviewing her, when we were going, I came to a certain question , when I asked her that question, (.) she said "No, why should I answer that question, that is about her mom". I I asking her, I didn't stopped at her, //mhm// but asking and asking about the parents, to why should I be asking, she cannot answer me. She send me away. //mhm// (.) Yeah, that one was very bad. #00:07:05-8#

I: And a especially good interview, that you remember? #00:07:08-5#

P: Especially good? That was the first interview I had, then I was not experienced, but the lady was so kind and so nice to me, she give me all that I need from her. #00:07:16-4#

I: Yeah #00:07:16-8#

P: Yeah (.). Very very good interview. #00:07:19-6#

I: What were the questions you found most difficult to ask? #00:07:23-8#

P: To ask, about their menstrual cycle #00:07:26-6#

I: Okay #00:07:27-0#

P: Yeah #00:07:27-4#

I: Ah, what do you feel the persons you were interviewing found most difficult to answer? #00:07:34-3#

P: Mind, what is difficult to answer? #00:07:36-7#

I: Yeah #00:07:37-0#

P: Yeah, (...) abortions. #00:07:40-7#

I: //Okay// #00:07:42-0#

P: //The// number of abortions they had. Because they don't even wanted the menstrual //mhm// ones. Yeah #00:07:45-0#

I: Ahm so we are nearly at the end, so I will just ask you some questions about you. Ahm, which ethic group do you belong to? #00:07:53-2#

I: So, do you want to add anything at the end? #00:08:04-7#

P: No #00:08:06-4#

I: Okay #00:08:06-4#
