# Supplementary material for: Gambian cultural beliefs, attitudes and discourse on reproductive health and mortality: Implications for data collection in surveys from the interviewer’s perspective
Source: PLoS One. 2019 May 16;14(5):e0216924. doi: 10.1371/journal.pone.0216924 (PMC6522014; doi:10.1371/journal.pone.0216924)
Supplement: S3 File — (ZIP) [file pone.0216924.s003.zip › S3_interviews/interview_811_0128.pdf]

Interview eight

Setting: Gambakunda, in a courtyard in front of a house of a respondent
Date: 16.03.2016
Time: 15:44
Total interview time: #00:11:27-9#

I: (.) Ah now I will ask you about you relationship with the community members. #00:00:26-8#

P: Yeah, the relation with the community member, is not that much difficult. Sometimes, we did it difficult, sometimes we can cope with it. It's just left to how we introduce ourselves with them. (.) So (.), but the time we were in Bakaday it was a bit difficult with us. Eventually there was/ we are having that problem, here in this in Dembakunda (.) kuta. Because, we went to our compound, one men came out, telling us that we people are just taking money from us, talking to us, you don't bring anything for us. I told him that "no, we are not (.) those people". He thought we were the government, the people issuing the ID-Card. I told him, "it's not the same we are under the MRC. And MRC we are dealing with the health, if you can know that", because the time we were introducing ourselves he was not there. //mhm// So when we came, the elderly (.) ah brother talked to him, but he didn't understand. Then I have to explain (inc.), but still (.) he was not comfortable with us, he said he said we should not talk to the ah to the wifes. Because we always come, asking question, to many question and we don't give them anything. We don't do anything for them. (.) And I told him, that (.) "not yet for what you people, but people are benefiting from the MRC, I don't know wherever you people were benefiting, but people are benefiting from it. But I don't know how you how come." A/ b/ by the time the brother told me, that "he was not here he was in Spain, he just came". //mhm// I told him "Many people are benefiting, I don't what about you people here, but it's what we are trying to do now." So that we can talk to the people and understand, and understand and know what is the essence of MRC. //mhm// (.) Yeah. #00:02:12-5#

I: So is it generally a good relationship to the community members would you say or a bad one? #00:02:18-9#

P: Is sometimes is good, but you know sometimes is not good with them. (.) Yeah. #00:02:23-5#

I: Ahm how did the community react on (.) ahm you your new responsibility? So #00:02:31-4#

P: Ahm when we are reaching them, when we talk to them, because when we are normally reach to a village, when normally go to the Alkali. //mhm// (.) So when we go to the Alkali, the Alkali will talk (.) will talk will call the villager call that, then the villager (inc.) have to help us, to find the people we are trying to get. (.) Sometimes the people we used to go are not, is difficult to cope with them. So some do respond, but some will never want to talk to us. (.) Yeah. #00:03:00-2#

I: Ahm, (.) What is you impression? #00:03:03-6#

P: (.) Yeaaah, (.) is good is is is ah good aah (.) till to interact with people and know peoples view, //mhm// what they want and what they don't want. (.) When they inter/ ah when they don't want to talk to us, we will convince them, (.) so they will want to, so they will want to understand what you are after. //mhm// Yeah, most of the time we do this. (.) Yeah. #00:03:27-7#

I: Ah, did your being a female (.) ah had have any influence on the responses of the community? #00:03:35-2#

P: Mh no, (.) not every where (.) //mhm//, more like some community (.) they were dress codes to matters, the way we dress (.), sometimes they will like it, sometimes they will not like it. But anyway we are going another, the this people is just stress for them life, so we normally have that kind of dresses on for them, so that they were able to talk to us. (.) Yeah #00:03:58-9#

I: Ahm, do you feel it is difficult for some women to tell you about their health information? #00:04:05-3#

P: Yes, sometimes it's difficult for them to talk to us (.) //mhm//. Yeah. Especially the people we cannot speak t the same languages. Language barriers (.) and we don't have somebody to interprate us, it's difficult with them. #00:04:18-0#

I: Yeah #00:04:18-5#

P: Yeah #00:04:19-6#

I: Ahm, why do you think it's difficult for them, if it is not the language? #00:04:24-6#

P: If I told them the language, we are not speaking. Is is not difficult, but if they cannot speak the language and there is no interpretator is not accepting for them. It's very difficult. Without an interpretator it is very difficult. (.) Yes. #00:04:33-8#

I: Now I will as you about you general fieldwork experiences. Ah, please tell me about your experiences during the fieldwork. #00:04:41-6#

P: Experiences ((laughing)), it's really difficult, more difficult. //mhm// Definitely, the the the most difficult one is (.) when we are coming to work, our food (.), the water we used to drink //mhm// (.) and when we the car, when we are coming to work, the car when we come, coming is not difficult, but when going, when the other one come before the other one comes for us (.) it's difficult. (.) Yeah. (...) This is normally a difficulty. (.) And you know we normally work up to Saturdays, Sunday only Sundays (inc.) we have, to wash our and items. Sometimes we don't even wash. Or a few people don't wash from us. Me especially for me. Because I don't have time. (.) Yeah. #00:05:31-3#

I: Ah, what do you think went well? #00:05:34-3#

P: (.) Went well. Between ourselves? //mhm// (.) Yeah it's fine, we understand each other very well. //mhm// (.) Yeah. So in the fieldwork is nice to meet people, (inc.) with people. (.) Yeah #00:05:48-7#

I: Ah, what were the //challenges?// #00:05:52-0#

P: Because, some some places we don't know them. But now we really knows, because we are Gambians, //mhm// but most places are in The Gambia, who we don't know them. But we be-because of this experience we know many places, and we meet sometimes good people. And they really appreciate us, definitely. //mhm// (.) Yeah. Definitely. The challenges are sometimes it's difficult with our languages. Especially this community, they are Sarahule, to it is very difficult for us. //mhm// (.) Yeah, it's only that. But me I can speak Fula and Mandinka and Wolof. (.) Yeah. #00:06:24-6#

I: Ah did you have any positive experiences? #00:06:28-7#

P: (.) Positive experiences (...), like with the community or with ourselves? #00:06:34-9#

I: With yourselves, with the community, in general. #00:06:37-9#

P: Positive, (inc., unclearly spoken). (.) Yeah, because with ourselves we understand each other, when we talk to each other. So in the community too, (.) they really appreciate it, definitely. (.) //mhm// Anyways (inc.) they appreciate it. It's only the language sometimes, (.) it's difficult (.) yeah. Because since I started it's only today I have this difficulties. (.) For someone to talk to someone here that "no you people are just to much, you don't come and you don't bring anything, just talk talk." It's only today, but is it's fine. It's very excellent. #00:07:13-2#

I: Do you have a suggestion how this could be solved? #00:07:16-8#

P: Yeah, maybe the they have to try to help us about car, the feeding and the water. (.) You know sometimes, you when you miss this water, it's a problem. (.) You have to go feeding, the water (.) yeah. //mhm// And the car for us, it's very difficult. #00:07:34-8#

I: (...) Can you remember the first and the last interview you performed? #00:07:41-4#

P: Yeah #00:07:42-2#

I: Can you describe them a little bit in the differences maybe between them? #00:07:46-9#

P: Yeah (.). That is today. The first day I was in the field (.), it was excellent. I meet a woman, the woman is an old woman, but she come and (inc.) me and she treated ah a her friend. (inc.) But for the last one today here, (.) the last compound here was (.) it was difficult with them //mhm//. Yeah (.) it was difficult. #00:08:08-9#

I: Ah what was an especially good and an especially bad interview? #00:08:13-4#

P: The interview I have (.), the good one (.) //mhm//, when I interviewed that women, the lady (.) it's was it was a young girl. After she asked me, wherever she can come to the MRC. I told her "Yes". (.) The last one I have, the good/ ah the bad one today. (.) Oh, I was so embarrassed. I really feel ashamed of, because I explained myself, (.) he cannot understand. I called somebody to for him to explain and talk to him, still the person cannot understand.

(.) I was really embarrassed. (.) Yeah. #00:08:48-5#

I: Ahm, what were the questions you found most difficult to ask? #00:08:55-3#

P: Pardon? #00:08:56-0#

I: Ahm the questions you found most difficult to ask the people? #00:09:00-0#

P: It's the menstrual cycle. #00:09:02-0#

I: Okay #00:09:02-2#

P: That is the one, the most difficult one. And the (.) natural mothers birth. (.) They will never want to tell you about it. Especial this Fula and the Sarahule community. (.) They will never want to tell you about and I don't know why. //mhm// Yeah, that one is very difficult for us. #00:09:20-7#

I: What questions do you feel, ah the respondents, so the people you were interviewing, ah found hard to answer? #00:09:28-7#

P: Their dead their dead ones. Yeah their dead children (.) it's difficult for them to say it. (.) Or the miscarriages (.) yeah. //mhm// So that one is difficult for them to say. The miscarriages they can say, but about their deaths, (.) especially the compound I went, this woman. The woman, the first four children were all dead, yeah (.), only one stillbirth, but the three alive borns there are dead. She did she didn't even want to tell me (inc.). //mhm// Yeah. #00:10:01-6#
