# Supplementary material for: Gambian cultural beliefs, attitudes and discourse on reproductive health and mortality: Implications for data collection in surveys from the interviewer’s perspective
Source: PLoS One. 2019 May 16;14(5):e0216924. doi: 10.1371/journal.pone.0216924 (PMC6522014; doi:10.1371/journal.pone.0216924)
Supplement: S3 File — (ZIP) [file pone.0216924.s003.zip › S3_interviews/interview_811_0130.pdf]

Interview nine

Setting: Gambisara, in front of a house, on a bench. In a backyard, many kids and women are around.
Date: 21.03.2016
Time: 12:38
Total interview time: #00:07:45-5#

I: Okay (.) ahm, now I will ask you some question about the relationship with the community members, you have been working with or or living with during the fieldwork. (.) Ahm how would you describe your relationship with the other members of the community? #00:00:44-0#

P: The where the place I am living? It's fine comp/ place. I live with my cousins brother, but it's fine. #00:00:51-3#

I: And the community in the fieldwork? #00:00:53-3#

P: Yeah, the community in the f/ it's hard in the fieldwork, because sometimes the language barrier is the problem. //mhm// And sometimes to get the information from people, is very difficult to have ther own information, private information. And their paper I mean the ID-Cards or the //mhm// passes is difficult to get get them from the people, is very hard. //mhm// #00:01:14-0#

I: Ahm, (.) so would you call it a good or e/ either a bad relationship in general? #00:01:20-0#

P: You know is it's not easy at all, it's hard. It's hard to get people information. //mhm// Private life it's not easy at all, it's hard. #00:01:27-5#

I: Ahm, how did the community react on your new responsibility? #00:01:32-1#

P: Ahm, yes when we start to ask them they they became surprised. (.) //mhm// But (.) in meantime they used to understand us. (.) Yeah. We are coping with them //mhm//. #00:01:43-7#

I: What is your impression? #00:01:45-6#

P: (.) Impression about ? #00:01:48-1#

I: The community, the work with them. #00:01:49-9#

P: Yes, it's cool. Is sometimes, it's used to be hard, because the sun, as you can see the sun. Sometimes we are, we used to be very tired, but we are managing. #00:01:58-3#

I: Did your being an female had a-any influences on the response of the community? #00:02:05-6#

P: Excuse me? #00:02:06-4#

I: Ahm, did your being female had any had any in-influence on the response ahm of the community? So did your being female has any influence on you acting with the community, that would that make a difference? #00:02:22-1#

P: No, (.) like I am a female? #00:02:25-0#

I: Mhm (agreeing) #00:02:25-3#

P: Yes is a difference, because as a men, when a men come to ask them, maybe they will not response them. But as a woman, it's used to be (.) easy //mhm// for them. #00:02:34-7#

I: Ahm, do you feel it is difficult for some wo-w-women to tell you about their health information? #00:02:41-6#

P: Yes, some womens if is if you ask them their parents, I mean the number of birth their mother have, (.) sometimes they re-refuse, they will not tell you the number of birth their m/ parents have. They say "No, it's not important for (.) me to tell you the number of birth my mother have." Is difficult for them to tell you that. And if you asked them, they used to be surprised. "Why are are you asking me that question?" #00:03:03-5#

I: Ah, why do you think it's difficult for them? #00:03:07-3#

P: Ahm actually you know, this people sometimes I think it's lack of education. If you ask them those kind of question, they become surprised, because they are not educated. (.) So that's what I think. //mhm// #00:03:17-4#

I: Ahm, are there certain people who find it more difficult, like young ladies, eldery women (.) or some people from a certain ethic group? #00:03:27-8#

P: Yes of course they find it difficult. Many of them, (.) any village we went, there are people who will find it difficult. (.) Yes. #00:03:35-4#

I: So now I will ask you questions about your f-fieldwork experience. (.) Please tell me about your e-experiences in the fieldwork. #00:03:44-1#

P: Ahm excuse me? #00:03:45-3#

I: Pleas t-tell me about your experiences in the //fieldwork// #00:03:48-7#

P: //Yes// my ex-experiences field is, I've never been here before and now I've been here with many people, with many tribe like Fulas and Serahules, before I have never been her, but now us, I am in the fieldwork, I have been in many villages. #00:04:06-0#

I: mhm (.) What do you think went well? #00:04:07-8#

P: What? #00:04:08-9#

I: What do you think went well? So what was good? #00:04:11-4#

P: What was good. //mhm// (.) In the field? #00:04:14-5#

I: Yes #00:04:15-0#

P: Mh (.) yes sometimes it is good to come in the community, because they (.) they welcome us nicely and they are happy to meet with us. (.) Yeah. (.) Is is a good thing. #00:04:26-7#

I: Ah, where were the challenges for you? #00:04:31-2#

P: Ahm, it was hard, when we are in Bakaday. It was hard when we are there, we sleep (.) down and there is no electricity (.) and it was hard there. We provide everything for ourselves. The food, the water, then there is no water, it was hard. #00:04:46-8#

I: Mhm (...) did you have any positive experiences? #00:04:51-5#

P: Yes just just what I just said right now. (.) Yeah. #00:04:55-9#

I: Mhm, ah did you have any negative experiences? #00:04:59-4#

P: (...) Ahm yeah, (.) it's difficult to stay outside as a f-female, it's difficult to stay outside. And when we are in Bakaday we feed ourselve and (.) the water is a problem there, there is no electricity, it was very hard to us to stay outside. #00:05:18-3#

I: Do you have any suggestion how this could be solved? #00:05:21-5#

P: Acutally, (.) maybe @(.)@ maybe there is a suggestion, if they provide the mattresses for us and the food also, it can be possible. @(.)@ #00:05:34-0#

I: Okay, do you remember the first and the last interview, that you performed? #00:05:40-1#

P: (.) Acutally I don't think I (...) Oh, I think I forgot the first interview.@(.)@ #00:05:49-1#

I: Okay (.) it's fine. Ahm what was an especially good and a especially bad interview? #00:05:56-7#

P: Ahm okay, if you ask them the menstrual side, (.) sometimes it's it's bad, (.) because they were not accepting us to ask them like that. (.) But in that part that one is not good. #00:06:06-9#

I: And the good one? #00:06:09-0#

P: The good one is when you ask them I mean about, wherever the any miscarriage or stillbirth or abortion, that part is the good one. Because they they thought that we will help it them in other way. #00:06:23-0#

I: Ahm, what were the questions you found most difficult to ask? #00:06:26-8#

P: That's the part, the (.) miscarriage, the abortion or the menstruation, //mhm// sometimes you feel ashamed to ask the woman, because she is older than you. You feel ashamed to ask her about that. (.) The menstruation side. #00:06:40-3#

I: What questions do you feel the respondents found most difficult to ha ha answer? #00:06:46-7#

P: Question? #00:06:47-6#

I: The people you were interviewing, what were was the question they found most difficult to answer? #00:06:53-3#

P: I mean, if you ask them the number of children their mother have, some it it is difficult for them to answer you, because they will start thinking, or counting //mhm// the number if birth their parents have. (.) Yeah it's a difficult one. #00:07:05-5#

I: So, we are nearly at the end. I'll just ask you some questions about you. (.) Ah what which ethic group do you belong to? #00:07:13-5#

I: Okay, is there anything you you want to add at the end? #00:07:25-3#

P: Ahm (.) just if they can help us (.) to provide something for us. Like when we went to the village to stay, the mattresses we are living in, is is very bad. And we provide food, everything for by ourselves. In that case, if they'll can help us, (.) that would be very good. #00:07:45-0#

I: Okay, thank you. #00:07:44-8#

P: Thank you #00:07:45-5#
