# Supplementary material for: Gambian cultural beliefs, attitudes and discourse on reproductive health and mortality: Implications for data collection in surveys from the interviewer’s perspective
Source: PLoS One. 2019 May 16;14(5):e0216924. doi: 10.1371/journal.pone.0216924 (PMC6522014; doi:10.1371/journal.pone.0216924)
Supplement: S3 File — (ZIP) [file pone.0216924.s003.zip › S3_interviews/interview_811_0131.pdf]

Interview ten

Setting: Gambisara, in front of a house, on a bench. In a backyard, many kids and women are around.
Date: 21.03.2016
Time: 13:01
Total interview time: #00:09:01-0#

I: So now I will ask you some question about ahm your relationship to the community members. How would you describe your relationship to the commu/ members of the community? #00:00:34-4#

P: Ahm, sometimes it's hard but sometimes also in the village it's easy. (.) Yeah because. Sometimes they are very hard. #00:00:45-6#

I: Stop for a second #00:00:45-0#

>> Interview stopped because of to laud background noise<< #00:00:45-0#

P: But if you manage also is easy you know, some people they are not easy to interview. And in the community, community members also. Some people if you just go, (.) for the first time, they will not, they will not comfortable you too at all. (.) Yeah. #00:01:00-5#

I: Is it a good relationship? #00:01:03-1#

P: Yeah it is good to (inc.) with them. (.) Yeah. #00:01:07-4#

I: How did the community react on you new responsibility? #00:01:11-3#

P: Yeah, sometimes easy, but sometimes also it's hard for the siblings and menstruation s-side, it's very difficult for them to answer, because some of them they are elder than you, they think you come and know their part. //mhm// (.) Yeah. #00:01:27-2#

I: What is your impression? #00:01:29-7#

P: Like? #00:01:31-3#

I: Ahm the impression of the community? #00:01:34-5#

P: My impression? #00:01:35-7#

I: Impression #00:01:36-0#

P: yah (...) some people they welcome you nice, but some they are very difficult. The moment you reach they before they understand what (inc.) they are, they will not welcome you easily. They will be hard with you, some people is that they are believe you, they will not welcome a stranger, (inc.) until they are used with you. And also we don't stay, (.) yeah just going, when we reach until when we are believing them, they thank you with a smiling face.

//mhm// (.) Yeah.   #00:02:10-4#

I: Did your being female had a-any influence on the re-responses of the comu- community? #00:02:16-4#

P: Yeah for the siblings they will not answer the thing that you come and ask. (.) Some of them, (.) they will not want, they will say " (inc., unclearly spoken) Why are you asking for the thing?". And some will say "My eldest some of them passed away, I will not tell". (.) That is it.   #00:02:33-1#

I: Do you feel it is difficult for some women to tell you about your health information? #00:02:39-4#

P: Yeah, is it's hard for them to tell you sometimes, because some of them, when they have child who passed away, the moment they want to tell you, is when they crying. Some of them they will tell you about, when they have the, they will not want (.) sometime. #00:02:56-1#

I: Why do you think it is difficult for them? #00:02:59-1#

P: Yeah you know some of them they loved their kids too much. (.) Some when, sometime when they are telling you, they will not want, because that one has passed away and they loved the child too much. They will not want to tell you, or before telling you, you see them, they chain their face and they cry (.) in time.   #00:03:17-7#

I: Are there certain people who find it more difficult than others?   #00:03:22-1#

P: Yeah, their, some of them (.) it's not easy at all, before they tell you that, and it's very difficult. (.) For them to tell you #00:03:34-9#

I: Ahm please tell me about you experiences during the f-fieldwork? #00:03:40-2#

P: Yeah, it's not easy, because the sun is hot, some some of them you will go there, treat them and re-visit, re-visit, re-visit them for the paper, they will not have. Some of them they will have, but they refused to give you, they think you will take their papers. Some of them think that you come and (.) interview them, that's the thing, some of them they think that, after (inc.) they will welcome you.   #00:04:05-4#

I: What do you think went well?   #00:04:07-7#

P: What do you think?   #00:04:10-2#

I: Went well, what was good.   #00:04:11-5#

P: Is good, some of them, when you ask them miscarriage and abortions, stillbirth, they will think that when you come and cure them. (.) And with the moment you some of them they think that you will come and give them medicine. (.) It's difficult.   #00:04:27-2#

I: What were the challenges? #00:04:29-2#

P: Ah the challenges is very hard, because (.) when the time when the time we went to Bakaday they don't have , the bed is very slight and is not deep. The moment you sleep there, when you wake up your chest hurt and (.) where we find water is very hard. (.) Yeah. #00:04:48-7#

I: Ahm (...), did you have any positive experiences? #00:04:55-4#

P: Yeah, (.) positive (.) yeah, is because were we are sleeping with the doors, is not correct. The door is not good at all. It's not good, because there is no (inc.), they will not lock you, you will just stay lying in bed and sleep and all of us are girls there, and the village we don't know. We are just managing that all. #00:05:23-8#

I: Did you have any negative experiences? #00:05:26-4#

P: Negative expression? No. #00:05:31-1#

I: Ahm, can you remember the first and they last interview you performed? #00:05:37-0#

P: Yeah, the first interview I performed was the practical time, the time we are they test us practical and the last one is today. The interview I did today. I did it on Saturday, but that time we don't have the papers after that I came to them and make it (inc.). That's why I don't at interview them, I interviewed on Saturday (.) but this people people they don't have the paper by that time the household member who I went out, that's why I don't enter it all the day. I went on today, so we have talent data. #00:06:10-6#

I: Ahm can you describe ahm the first/ the different experiences between them? Between your first and the last interview? #00:06:18-6#

P: Yeah, the first is was difficult, because the person I met, that one is a business women (.) the time I went in they welcomed me, but the time I was asking the question, too long it say "Had I know it it would stay like this, I would not welcome you. Because it take to much of time." (inc., unclearly spoken). And on Saturday I interviewed this one, it was kindly welcomed, it was nice the woman was nice (.) I interviewed. #00:06:47-5#

I: Ahm, what was an especially good and and especially bad interview you performed? #00:06:51-5#

P: Yeah, the good thing was, when we were interviewing them, the first one the first one I interviewed was, (.) 45 or 46 years, it was difficult the time asked her the menstruation. And if have kids that are elder that you. And this one, maybe the first child is seven years (.) five, that's why it is different. #00:07:15-3#

I: Ahm, what were the questions you found most difficult to ask? #00:07:18-7#

P: It's the (...) menstruation and the sibling and abortion. Even some of them, they will have it, but they will not tell you, because in our society, it's not good, to tell you the abortion.

Even if you have it, the mother will hide from you , that you don't have it, even when interviewed. And some of them, they will have child without having husband, when we are coming to list the household (.), sometime if you don't ask properly, you will not get that child, because is/ the the girl have it without husband in our society, they wont they don't want, they used to hide that. They will not tell you, (.) that's why. #00:07:58-3#

I: What questions do you fell the respondent found most difficult to answer? #00:08:05-2#

P: Is the sibling and the menstruation and abortion. Miscarriage and stillbirth they answer you, (.) yeah. But Menstruation (.) still/ ah abortion and still/siblings, they find it difficult to answer. #00:08:22-0#

I: Okay, we are nearly at the end, I just want to ask you some questions about yourself. Ah, which ethic group do you belong to? #00:08:29-4#

I: Is there anything that you want to add at the end? #00:08:59-4#

P: No #00:09:01-0#
