# Supplementary material for: Gambian cultural beliefs, attitudes and discourse on reproductive health and mortality: Implications for data collection in surveys from the interviewer’s perspective
Source: PLoS One. 2019 May 16;14(5):e0216924. doi: 10.1371/journal.pone.0216924 (PMC6522014; doi:10.1371/journal.pone.0216924)
Supplement: S3 File — (ZIP) [file pone.0216924.s003.zip › S3_interviews/interview_811_0132.pdf]

Interview eleven

Setting: Gambisara, at the back of a house, on a kind of veranda. Sitting on two chairs. Not much background noise. No people in the closer area.
Date: 21.03.2016
Time: 13:40
Total interview time: #00:08:59-6#

I: Okay, now I will ask you some questions about your relationship with the community members. Ahm, how would you describe your relationship with the other com- members of the community? #00:00:41-3#

P: That the work we are doing with? #00:00:43-9# #00:00:43-9#

I: Mhm (agreeing) #00:00:44-3#

P: Some, are they our relatives, but some I have never been to that places. The places that we are visited, I have never been gone to this places. But some villages are there, some of our relatives are there. (.) Yeah. #00:00:58-7#

I: Is it a good relationship? #00:01:01-5#

P: Mh? #00:01:01-8#

I: Is it a good relationship to they community? #00:01:04-1#

P:Yeah, mhm. #00:01:06-9#

I: Ahm how did the community react to your new responsibility? #00:01:10-9#

P: Pardon? #00:01:12-9#

I: How did the community react to you new responsibility? #00:01:17-1#

P: (inc., unclearly spoken) Some of them are very re/ ahm how to call it? (.) Are very friendly. They are in other words some, the way you approach to them //mhm// the will also react to you in that way. If we approach to them in a good manner they will, they will also respond to you in a good manner. (.) Yeah. #00:01:44-4#

I: Ahm, what is your impression? #00:01:47-1#

P: Abou this, OH I am a Gambian, I never knew that some villages are around this area. I have got a very good experience. As as a Gambian, knowing all this villages, (.) be I have never been to those villages, but now I know very very well villages and I have the experience of being a fieldworker. //mhm// (.) Yeah. #00:02:08-0#

I: Ah, did your being a female (.) have any any influence on the the re-responses from the community? #00:02:17-8#

P: No #00:02:18-3#

I: No #00:02:18-6#

P: No #00:02:18-9#

I: Okay, do you feel it is difficult for some women to ask ahm to answer their questions about their health information? #00:02:26-9#

P: Some yeah (.) mhm. #00:02:29-3#

I: Why do you think it is difficult for them? #00:02:31-2#

P: Some, if you tell them some will feel sad of it will remind them of past events some like for example, when the persons child died, if you ask that person all miscarriages, stillbirths it will remind them about those lost, it will a difficult. It is a difficult one. #00:02:47-5#

I: Are there certain people who find it more difficult that oth-others? #00:02:52-8#

P: Yes! Because some people they will not even respond to you in (inc.), even if you clear them, some will not respond to you. (.) Yeah #00:03:03-0#

I: Ahm, please tell me about you experiences in the field wo-work. #00:03:07-4#

P: Ah (.) experiences. Yeah experiences I remember about our feeding. Feeding is the one major problem. //mhm// (.) And the what they are paying us, really is not satisf/ satisfying for some of us. If can see maybe you are responsible about for your father and your mother, you have (inc.) if you had to buy a bag of rice and when you go to a particular village we are supposed to feed yourself. Breakfast ahm (.), dinner, lunch and you (inc.) have to to afford that from that payment, the rest of it, you're your don't left anything. //mhm// (.) Yeah. That's the problem with that. #00:03:47-1#

I: What do you think went good? #00:03:49-7#

P: Mhm? #00:03:50-3#

I: What do you think went good during the //fieldwork//? #00:03:53-5#

P: //Ah// for me g/ for me The experience I am getting, that one is extremely good for me. (.) yeah #00:04:00-7#

I: What were the challenges? #00:04:03-6#

P: Challenges? (.) Yeah for example, as I told you earlier, (.) some to find to look at their households, sometimes are (inc.), for example you going to those the villages, if you go there while the sun is hot, if you go and ask them (inc.) ask them for this woman, asking for this men or woman, some people they wont even look at you, as if you are a woman //mhm//

(inc.). Mhm. #00:04:27-6#

I: Do you hav/ did you have any positive experiences? #00:04:31-9#

P: Mh (.) No #00:04:34-3#

I: Mhm, (...) did you have any negative experiences? #00:04:37-7#

P: Mh (...) No, I don't know. #00:04:42-4#

I: Okay (.) mhm, (.) can you remeber the first and the last interview you you performed? #00:04:49-9#

P: The first one ? (...) The first one was in at (.) mhm at Basse //mhm// the persons name is ahm (.), was it [...]? I forgot the name //mhm// right now here, is ahm (.) [...]. //mhm// (.) Yeah. #00:05:08-6#

I: Can you describe (.) ahm the difference between the interviews? (.) Between your first and the last interview? #00:05:16-0#

P: The first it was bit as it was bit bit complicated. But now (.), oh is @(.)@ you will think first you will think difficult, but (inc.), but as time goes one, you will know everything is simple than yourself. #00:05:29-9#

I: What was an especially good and an especially bad interview? #00:05:35-3#

P: Pardon? #00:05:36-5#

I: An especially good and an especially bad interview you performed? #00:05:40-5#

P: Especially good (...). Oh okay, (...) when you come to interview a person and a a person, when you ask the person about what you want, you if you introduce yourself for the purpose of you doing that, (.) you tell ask the person, the t/ (.) ahm about the questions that you want and if the person response you well, response all the things that the questions require to the answers, (.) that one is a good experience. //mhm// (.) But if you ask someone, (.) the person they will respond you, res/ restfully, oh! That one is a negative //mhm//. (.) Mhm. #00:06:24-1#

I: Ahm (.), what were the questions you fo-found most difficult to ask? #00:06:30-1#

P: The questions, the f/ about miscarriages, if you to ask them they don't (inc.). Some people correcting you about miscarriage, they don't want. It will be very difficult for some of them. You will (inc.), but some of them, it will be def/ very difficult to tell. (.) Yeah #00:06:48-1#

I: What questions do you feel the respondents found hard to answer? #00:06:53-6#

P: Mh, (...) which one, (.) is/ ah okay (inc.). (...) Ahm, (.) if ah you ask the person about the (inc.) mortality of the person, they will tell „(inc., unclearly spoken) I don't know how many

children my mother have. I don't know" You will ask (inc., unclearly spoken), you will beg them, some will answer you (inc. unclearly spoken), that one is (.) yeah too bad. #00:07:29-3#

I: At the end, is there anything that you want to add? #00:07:50-4#

P: Mh (...) like what they are paying us for it, it it doesn't satisfy us. //mhm// At the and if you payed the money (inc., unclearly spoken), after we've payed I have to buy bag of rice, (.) then when we will go to this villages, it will cost for living in this those villages. Very difficult, feeding is a problem, oh yes. (.) Yeah, feeding problem. (.) Mhm. #00:08:20-8#

I: Do you have any suggestion how this could be solved? #00:08:25-1#

P: Ahm, (...) If there is possibility, in a way that the can help us, (.) with our feeding, even if they could increase our sellerie for us, if they can provide like, rice oil any like, so that when we are going to the villages, (.) we take it with/ You know when we are in Basse here, there is no Problem, after work we are going back to our place. But when we go to those villages, (inc.) it is another thing. And that (inc.) when you go to those villages , they will be no light, you have to pay ten Dalasis or 15 Da-Dalasis in order for you to charge your phone. Oh #00:08:57-1#

I: Okay #00:08:58-4#

P: Yeah @(.)@ #00:08:59-6#
