# Supplementary material for: Gambian cultural beliefs, attitudes and discourse on reproductive health and mortality: Implications for data collection in surveys from the interviewer’s perspective
Source: PLoS One. 2019 May 16;14(5):e0216924. doi: 10.1371/journal.pone.0216924 (PMC6522014; doi:10.1371/journal.pone.0216924)
Supplement: S3 File — (ZIP) [file pone.0216924.s003.zip › S3_interviews/interview_811_0133.pdf]

Interview twelve

Setting: Gambisara, at the back of a house, on a kind of veranda. Sitting on two chairs. Not much background noise. No people in the closer area.
Date: 21.03.2016
Time: 13:55
Total interview time: #00:07:37-9#

I: Ahm (.) how would you/ now I will ask you some questions about your relationship with the community members you have been working and doing the fieldwork. (.) Ahm, how would you describe your relationship with the other members of the community? #00:00:47-1#

P: Like my co- workers or the community? #00:00:50-5#

I: The community you have been working in #00:00:52-1#

P: Yeah it was nice anyways, (.) yeah. (.) I met so many people, it was a good experience anyway. yeah. #00:01:01-8#

I: Ahm how did the community react on you new new responsibility? #00:01:06-5#

P: Like the interviews? #00:01:08-3#

I: Mhm (agreeing) #00:01:08-9#

P: I was fine. (.) Sometimes ahm ah difficulties, but (.) on my side it was okay. //mhm// Yeah. #00:01:19-6#

I: Ahm, what is you impression (.) about the community, about they have been reacting to you? #00:01:26-3#

P: Yeah I feel good. (.) Yeah. (.) It was okay. #00:01:31-7#

I: Did your being being a female have have any influence on the responses of the community? #00:01:37-8#

P: Like the? #00:01:39-7#

I: Ahm (.) this question here. #00:01:45-0#

P: (...) No #00:01:46-9#

I: No #00:01:47-5#

P: No #00:01:47-9#

I: Okay good. Ah, do you feel it is difficult for some women to tell you about their health information? #00:01:54-0#

P: Health information of others? #00:01:56-6#

I: Mh, of of the interviews you have been doing. Was it difficult for some people to tell you about their health information? #00:02:03-6#

P: Yea hm it's difficult for some to tell you about their (.) like pregnancies, their miscarriages and other stuff. Yeah. #00:02:13-1#

I: Why (.) why do you think it is difficult ? #00:02:16-1#

P: I don't know, maybe they don't want to share it with us, so we don't have to force them. //mhm// (.) Yeah. #00:02:23-4#

I: Ahm are there certain people who find it more difficult than others? Like young woman or elderly woman? #00:02:31-3#

P: Elderly, //mhm// yeah. #00:02:32-7#

I: Ahm, please tell me about your experiences during this fieldwork #00:02:38-1#

P: Yeah (.) we have some difficulties in Bakaday, //mhm// yeah, it was hectic there (.) the food and the water (.) yeah. And the room we were, the door @(.)@ it has no lock, //mhm// yeah, we we had a very difficult time there. #00:03:01-6#

I: Ahm, what do you think went well? (.) What was good? At the //fieldwork?// #00:03:13-4#

P: //At the villages// #00:03:13-1#

I: Yeah, with the //field // #00:03:14-6#

P: //in the field// #00:03:14-6#

I: Yeah #00:03:15-9#

P: Okay (.) #00:03:17-5#

I: What was good? #00:03:18-9#

P: @(.)@ It was good meeting people, knowing how they live, because this is my first time, (.) seeing some people living in (.) bad condition in (.) I don't know. Some of them, how they live it's (.) yeah it's not good anyways. (.) Yeah. #00:03:39-1#

I: What were the challenges? #00:03:41-1#

P: Challenges in the field? #00:03:43-6#

I: Mhm #00:03:44-7#

P: You know (.), it was okay though because we travelled, (.) the the car took there then bring us back. (...) It was okay anyways. #00:03:56-6#

I: Did you have any positive experiences? #00:04:00-2#

P: There? In the field? No. #00:04:03-9#

I: Did you have any negative experiences? #00:04:07-1#

P: @(.)@ Negative? #00:04:08-8#

I: It's no problem if you repeat yourself. (.) Just #00:04:13-2#

P: Yeah it's a good experience anyways, (.) I have known many places through this field fieldwork. (.) Yeah. Because, I have never been to such communities, this is my first time //mhm//. (.) Yeah. Is good anyways, it's a good experience. (.) Yeah. #00:04:33-9#

I: And did you have negative experience as well? #00:04:36-8#

P: Mhm @(.)@ no. #00:04:40-7#

I: Can you rem- remember the first and the last interview you performed? #00:04:49-5#

P: The first, like the first day? #00:04:53-8#

I: The first interview you performed? #00:04:56-6#

P: Yeah, that was in Basse //mhm//. Yeah #00:05:01-0#

I: Can you tell me about it? (.) How it was? #00:05:04-1#

P: @(.)@ You know it was okay, //mhm// no problems there, yeah. I was welcomed in the family so they answered everything I asked them. //mhm// Yeah. #00:05:15-2#

I: And the last interview? #00:05:16-9#

P: The last interview, like today? #00:05:20-0#

I: Yeah #00:05:20-9#

P: Yeah it's okay, no problem, the same thing (inc.) here #00:05:23-0#

I: Yes? So there was no especially difference between them? #00:05:27-0#

P: No #00:05:28-0#

I: Okay (.) #00:05:28-1#

P: The household head a ah welcomed us and gave us all the information we needed //mhm// from him. (.) Yeah. #00:05:35-2#

I: What was an especially good and an and an an especially bad interview? #00:05:41-3#

P: Like here? #00:05:45-8#

I: Mhm (agreeing) #00:05:46-4#

P: Today? #00:05:47-2#

I: No not today, in general. What was especially an good interview and an especially bad interview? #00:05:53-7#

P: Yeah ahm (.) some (...) some women like to answer all our questions. //mhm// So if we go to some certain point, they wont say "no I can't answer anymore". So they will leave, so we had to stop the interview. //mhm// Yeah. (inc.) (...) So because we cannot force them, to go for further. //mhm// Yeah. They are not willing. #00:06:18-7#

I: What were the questions you found most difficult to ask? #00:06:23-6#

P: About their menstrual (.) thing, their period. //mhm// To ask them. (...) Yeah some women like to answer that question, they will say "no, you cannot ask me about my menstrual thing"//mhm// #00:06:42-7#

I: Ahm, what questions do you feel the respondents fe/ feel most difficult to answer? #00:06:49-5#

P: Yeah, (.) That's the menstrual //mhm// thing. And their (.) if you ask them about their pregnancies, like this their miscarriages and stillbirths. Some women answer, and some say "no you don't have to know that" //mhm//. (.) Yeah #00:07:06-0#

I: Okay, at the end is there anything that you want to add? #00:07:23-1#

P: @(.)@ No, is good anyways, is a very good experience, knowing these villages and homeless because it's my first time. I am enjoying it. //mhm// Yeah, I enjoy the work. #00:07:36-3#

I: Okay #00:07:37-2#

P: Mhm #00:07:37-9#
