# Supplementary material for: Gambian cultural beliefs, attitudes and discourse on reproductive health and mortality: Implications for data collection in surveys from the interviewer’s perspective
Source: PLoS One. 2019 May 16;14(5):e0216924. doi: 10.1371/journal.pone.0216924 (PMC6522014; doi:10.1371/journal.pone.0216924)
Supplement: S3 File — (ZIP) [file pone.0216924.s003.zip › S3_interviews/interview_811_0134.pdf]

Interview thirteen

Setting: Gambisara, in a tailor's room. Standing opposite each other. Loud background noise from the tailor and people outside.
Date: 21.03.2016
Time: 15:27
Total interview time: #00:14:02-9#

I: Yeah, (.) no I will ask you about your relationship to the community members. #00:00:37-1#

P: Yeah it's alright and ah (.) on situational hand some people are a little bit okay, but you know it's different. (.) You know it's not everyone home who seems to willing and then cooperative doing this work. You know some people are so difficult at time to co_ to cooperate with us. You know there are certain questions, if you ask them they feel so bad, I mean they feel otherwise if you ask them, so (.) it's just something how to maintain I mean, (.) lot lot lot my patients to get what you want. //mhm// Yeah. #00:01:09-2#

I: So it's/ is it a good relationship? #00:01:11-8#

P: Yeah yes some how some how, yeah some how it's a good relationship, because you know (.) it's like and an advantage ah a adventure sort of (.) yeah. You know encountering difficulties and then (inc.). Also, (.) you know interacting with people, with different environments, areas where we have never been to. (.) Yeah especially here, (.) I've never been to here so, it's a (.) an experience for me, it have been one. //mhm// Yeah. #00:01:36-3#

I: How did the comu_ community react on your new responsibility with the interviews? #00:01:42-9#

P: Yeah some people, you know, react ah (.) well. (.) You know they appreciate it so much, therefore coming to us, then kissing. Some are so welcoming, (.) but then some (.) are a little bit pos_ negative. (.) But then we still, you know, (.) you know take it good faith, because (.) we ah (.) there for them, but then we are there for something else. (.) So you know we work so much hard, and then with so much patients (.) to get what we want. (.) Yeah. #00:02:11-0#

I: Ahm (.) what is your impression? #00:02:14-3#

P: Yeah, I am impressed you know (.) is like (.) it's like, (.) it's so educating. (...) I am edu_ you know it's educative (.) yeah, because I am experienceing so much good things about it. (.) You know it's like it's teaching me, (.) about so many things, that I never knew. (.) Yeah. #00:02:32-5#

I: Ahm (.) did your being a female have any influence on the responses of the community? #00:02:39-5#

P: Mh no. Not really //mhm// (.) yeah. #00:02:43-2#

I: Did you feel it's difficult to some women to tell you about their health information? #00:02:47-4#

P: Yeah, it is. Some people, like ahm the part of their ah menstrual period area, (.) if you ask them, (.) now some people (.) do not want to say, (.) you know (inc.). But some feel shy, (.) but //mhm// you know to me, I always talk to them like, they are woman and I am a woman as well, (.) so I don't think there anything wrong with asking them. So I always told them that, "even myself I go to that same (.) you know stage. (.) So (.) I am just appealing for you to (.) you know, ask/", how do you call it? "Cooperate with me and tell me this you know answers. Because I am a women like you"(.) yeah. #00:03:23-1#

I: Ahm please tell me about your experiences during this fieldwork. #00:03:28-4#

P: Excuse me? #00:03:29-4#

I: Please tell me about your experiences during this fieldwork. #00:03:32-8#

P: Yeah yeah yeah yeah, there are so many experiences. You know (.) experiences like, ahm (.) ahm travelling, like like working, going, coming. You know sometimes it's a little bit, (.) because we have ah the the our blocks are sometimes you know so far form each other, (.) like from one compound to another, you know, (.) though (.) it's a sort of exercise, but sometimes it's so tiering (.), because you have to walk from one compound to another, (inc.) even though there are so far far far away. Than secondly some people are never willing to come to us and tell some self- questioning. (.) Like sometimes, you know, there are people who will know, will resist do our questions, who will say "no, we are not interested" or " we don't have time, (.) you know, for you questions". Or we go to a particular place and there we start our interview, to half stage, you hear them tell us that, you know, (.) ahm "we don't have time, your questions are too much. We have some so so many things to do, so we are going". (.) They'll just go and leave us. And then sometimes it's twelve, it's to hot when it comes to to up once, it's too hot. (.) And then, (.) then then we are appealing, (.) because food is our our very important. (.) You cannot walk with an empty stomach, empty stomach. Sometimes we spent the our the little we have, (.) the little we are paid here, we spend it all for our food. Know so, it's a very very very very thing, that is disturbing us in times of ah the work. Because, if we are help at least, (.) like when we are based in a village, if they help with food. that's very important, because, if we had to send the little we had and to fe_ to feed ourselves, (.) what is the ah (.) the we are loosing at lot. (.) Is like we're just wasting so much energy and time. (.) Yeah. #00:05:21-8#

I: Did you have any positive experiences? #00:05:25-5#

P: Mhm, (.) no no no, not really. #00:05:30-6#

I: And negative? #00:05:31-8#

P: Yes! (.) You know (.) yeah, negative in ahm (.). We just need ah (...) ahm help from them, because if we had work and, (.) because we really want to do a good job for them. (.) We

really want to do that, because they are spending a lot on this, so we don't want to know you know, give them fake, you know work and any like, so we will be not fair to them, so we want them to f_ be fa_ fair with us as well. We want us to help each other, to have this motional understanding. Let them help us, let let us help them, because they want something very accurate and we are here to do that for them. But there is provided, that they are also trying to help us, because we are all the same. So let th_ let them help us, and we help them into (inc.). This is what we are asking of them. #00:06:21-0#

I: Ahm di_ do you have any suggestion, how this problem could be solved? #00:06:26-8#

P: Yeah (.), the only the only way that can be solved is to (.) the the hire administrative. (.) [...] and her team, they are the only ones who can help us, because they are the ones we are here for. We are working for them. (.) but then we are naive, because there are certain things we wo_ want to do for ourselves, but we cannot do it. (.) So we want [...] and the group and the others to help us sort out these things, (.) there are the only ones who can help us to sort this out, because we are here for them. So we are appealing, (.) it's not like the first, but we are apealing, (.) you know, for them to do something for us. Yeah, something so/ something very positive, you know which you think/ which we think will make our work easy and faster, (.) I think yeah. #00:07:13-5#

I: Ah, can you remember the first and the last interview you performed? #00:07:18-4#

P: The first and the last interview? #00:07:20-5#

I: Yeah #00:07:20-8#

P: Yeah, I can visibly remeber. //mhm// Yes, I (.) had an interview (.) ah 42 (.) one 42 (inc.) (.) and I interviewed a woman called (.) ahm [...] yeah, I interviewed her this afternoon. (.) Yeah. #00:07:38-2#

I: Ahm were was the difference between the first and the last interview you performed? #00:07:43-7#

P: Yeah yeah the first and the last, the first was, I was not much familiar with the work, (.) yeah. It was a little bit hectic for me, but now this last interview, I am so much familiar with the work, to understand that, you know, it's so easy for me to work and then question my question ah (.) interview. //mhm// (.) Yeah. In a s_ in a very simple way, because already used to it now. (.) Yeah. #00:08:06-9#

I: Ah can you des_ ah no. Ah what was an especially good and an especially bad interview? #00:08:14-5#

P: Ahm, yes (.) it was a, it was a (.) especially good one. (.) Because she was, you know, cooperative with me, she was not difficult and any question that I happened to answer. The other thing was their language barrier, (.) she was she speaks Serahule and I speak F_ ahm (.) Fula and Mandinka //mhm//, so I had to get someone, (.) you know, I had to ask for her consent, if there are certain questions, which you know that they have to ask her, (.) but I can't speak (.) the her language, neither she can speak mine. So if there is anyone whom she

trusts, who might sh_ within that place who can a_ who can help me, you know, talk to her, (.) for me to get that information, because is confidential, (.) you can not just go and ask somebody. (.) You know, (.) this is what I want, you oh you go and tell somebody, (.) I want you to talk to somebody and for her to tell me, what what's this going to, is confidential, is like you exposing her. (.) So, if I had to take her, ask for her consent (.) when she admit to for someone to help her during the interview. Then we called the lady and we she help do the interpretation. (.) Then when I the llllllady the lady talks to her, (.) then she respond back. (.) Yeah. #00:09:23-4#

I: Okay, ahm what were the questions, you found found most difficult to ask? #00:09:29-0#

P: Yeah (.), the period area (.) the period area is a very very difficult area to ask. The period area, because, you know, (.) some women, you know, and elderly women (.) they are more or less to be our mothers, (.) so you cannot, you know, you sometimes you feel so locked hand, (.) you just come directly to them and tell them that (.) "When last did you see a menstrual period?" Sometimes, like wearing, if they're were, if she is there with her children, (.) elderly children like us, you cannot just confront them and tell them "When last did you see your menstrual period?". So you feel locked hands, you feel sad and you feel so (.) how to call it? How, so you feel so (inc.) to ask her directly, (.) but you know, you have to make su_, but you have to ask her in anyway, (.) but then there is a way of asking her. (.) You know, like you (.) you can (.) "Sorry, mam there is that question that I want to ask you, but you have to forgive me". Because some people if you cannot, if you just jump and ask her a question she may think that you are acting rude, so you have to appeal first, then ask her that question. The period area is big (.) difficulty //mhm//. (.) Yeah. #00:10:38-6#

I: What questions do you feel the respondent found most difficult to answer? #00:10:43-4#

P: Yeah, the period area and their the number of natural children their mother has. (.) //mhm// Yes, and their miscarriages, (.) some they are stillbirths, they (inc.) ah forget the date, (.) the date of their miscarriages, so it's very difficult for you to capture those dates. (.) It, you know, requires so much (inc. (probit)) for you to get the accurate (.) answer from them. (.) Some people feel, you know, feel relocated to tell you how many children their mother has. (.) You have to ask her and ask her and ask her and ask before they agree. (.) Yeah. #00:11:15-8#
